# Individualized mRNA vaccines evoke durable T cell immunity in adjuvant TNBC

**DOI:** 10.1038/s41586-025-10004-2

**Published:** 2026-02-18

**Authors:** U. Sahin, M. Schmidt, E. Derhovanessian, A. Cortini, I. Vogler, T. Omokoko, E. Godehardt, S. Attig, S. Newrzela, J. Grützner, N. Bidmon, S. Bolte, S. Brachtendorf, T. Stuhlmann, D. Langer, D. Brüne, J. Blake, A. Feldner, H. Lindman, A. Schneeweiss, M. Eichbaum, Ö. Türeci

**Affiliations:** 1 BioNTech Group, Mainz, Germany; 2 TRON, Mainz, Germany; 3 HI-TRON Mainz, Mainz, Germany; 4 Department of Obstetrics and Gynecology, University Medical Center of the Johannes Gutenberg-University, Mainz, Germany; 5 Department of Immunology, Genetics and Pathology, Uppsala University Hospital, Uppsala, Sweden; 6 Division Gynecologic Oncology, National Center for Tumor Diseases, University Hospital and German Cancer Research Center, Heidelberg, Germany; 7 Klinik für Frauenheilkunde und Geburtshilfe, Helios Dr. Horst Schmidt Kliniken Wiesbaden, Wiesbaden, Germany

**Keywords:** Breast cancer, Tumour immunology

## Abstract

Triple-negative breast cancer (TNBC) is frequently associated with metastatic relapse, even at an early stage^1^. Here we assessed an individualized neoantigen mRNA vaccine in 14 patients with TNBC following surgery and after neoadjuvant or adjuvant therapy. In peripheral blood of nearly all patients, high-magnitude, vaccine-induced, mostly de novo T cell responses to multiple neoantigens were detected that remained functional for several years. Characterization of individual patients revealed that a large proportion of these T cells developed into two subsets: a late-differentiated phenotype with markers indicative of ‘ready-to-act’ cytotoxic effector T cells, and T cells with a stem cell-like memory phenotype. Eleven patients remained relapse-free for up to six years post-vaccination. Recurrence occurred in three patients: the individual with the weakest vaccine-induced T cell response relapsed, but achieved complete remission on subsequent anti-PD-1 therapy; another patient had a tumour with low major histocompatibility complex (MHC) class I expression with MHC class I-deficient cells growing out under vaccination; and the third patient was BRCA-positive and had a recurrence from a genetically distinct primary tumour. These findings demonstrate the feasibility of individualized RNA vaccines in TNBC, document persistence of vaccine-induced, functional neoantigen-specific T cells and provide insights into possible immune escape mechanisms that will guide future approaches.

## Abstract (web summary)

In a phase 1 trial, personalized mRNA vaccines tailored to individual tumour mutations in triple-negative breast cancer induced robust, long-lasting T cell responses and improved prognosis.

## Main

TNBC tends to recur and metastasize even at early stages of disease, with the recurrence rate peaking after about three years and declining rapidly thereafter^1^. DNA repair deficiency, intrinsic genomic instability and an immunogenic tumour microenvironment make TNBC an interesting candidate for individualized vaccination with somatic cancer mutations that can act as neoantigens^2,3^.

**Fig. 1:**
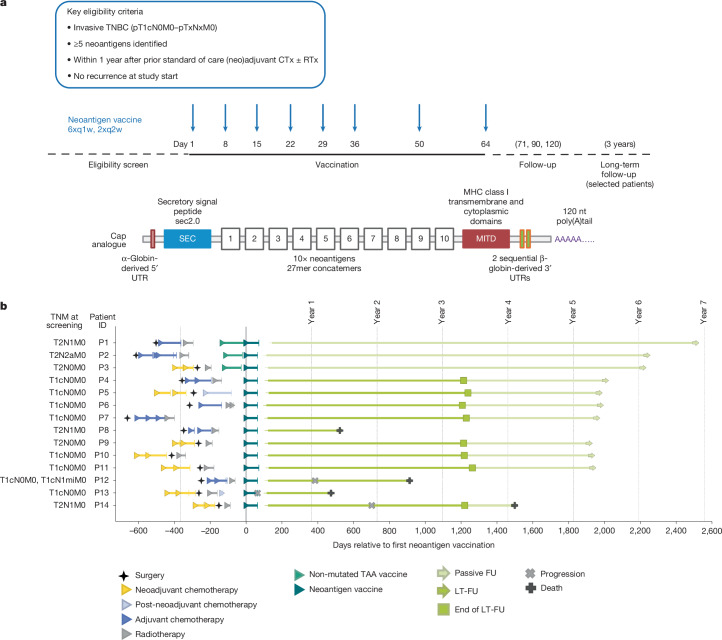
Trial design, RNA vaccine design and clinical course of patients with TNBC. **a**, Top, clinical trial design. Patients with early TNBC after surgery and neoadjuvant and adjuvant chemotherapy (CTx), with or without radiotherapy (RTx), were eligible within one year after having received standard of care treatment. Middle, a run-in cohort (*n* = 3, intra-patient dose escalation: 14.4 µg, 29 µg and 50 µg, followed by 5 × 50 µg) preceded the main cohort (*n* = 12) with patients dosed with 8 × 50 µg mRNA–LPX vaccine. Patients in the run-in cohort received pre-treatment (off-the-shelf mRNA–LPX vaccine assembled according to the tumour expression profile of the patient from a pre-manufactured warehouse of eight non-mutated TAAs) as a bridge until their personalized vaccine was available. Bottom, the individualized neoantigen mRNA vaccine consisted of 20 neoantigen vaccine targets encoded on two poly-lipoplexed mRNA molecules. For SNVs, the 27mer peptide region with the changed amino acid in the centre was used, and for frameshift-inducing insertions or deletions the sequence from the changed amino acid to the next stop codon was used. Open reading frames encoding the neoantigen target concatemers were flanked by a secretory signal peptide (SEC) and an MITD for enhanced HLA presentation. The 5′ and 3′ untranslated regions (UTRs) and the poly(A) tail designs of mRNAs were optimized for stability and translational efficiency. The three patients pretreated with bridging TAA vaccine were followed up passively. **b**, Prior treatment, trial regimen and disease course of patients enrolled in this trial. Out of 15 consented patients, 1 was discontinued after 3 vaccinations owing to TEAEs (hypotension, grade 3; nausea, grade 2; chills, grade 1) and is not displayed here. FU, follow-up; LT-FU, long-term follow-up; q1w, once a week; q2w, twice a week; TNM, tumour, nodes and metastasis grading system.

**Extended Data Fig. 1:**
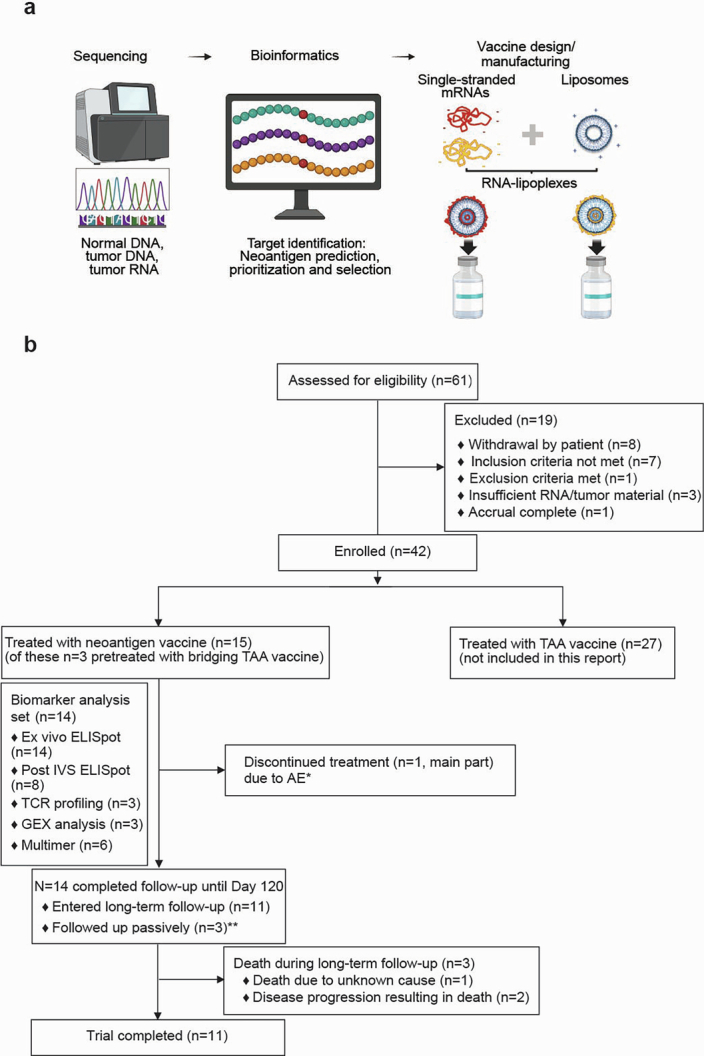
Manufacturing process and disposition of patients. (**a**) Schematic of production process. Schematic contains elements from ref. ^51^ (AAAS). Created in BioRender. Finlayson, A. (2025) https://BioRender.com/3lb1u3z. (**b**) CONSORT Diagram * Patient required a dose modification (total RNA) from 50 μg to 29 μg of the neoantigen vaccine due to treatment-emergent adverse events, and subsequently discontinued treatment after a total of three vaccinations. ** Beyond the protocol-specified follow-up period, the three patients pretreated with bridging TAA vaccine were followed-up passively; one patient consented to provide blood samples via a research project. ELISpot, Enzyme-Linked Immunospot (assay); GEX analysis, T-cell gene expression analysis; IVS, in vitro stimulation; TAA, shared tumor-associated self-antigens.

We developed an individualized neoantigen RNA vaccine approach that uses next generation sequencing (NGS) for mutation calling and predicts potentially T cell immunity-inducing neoantigens. This approach is being studied in patients with melanoma and pancreatic and other solid cancers^4–7^ (Extended Data Fig. 1a). To create the vaccine, multiple cancer mutations from an individual patient are concatemerized and encoded on two strings of immunostimulatory, non-nucleoside-modified uridine mRNA. Molecular designs of the cap, 5′ and 3′ untranslated regions, and the poly(A) tail of the mRNA enhance translation of the coding sequence in human dendritic cells^8,9^. A secretion signal and an MHC class I trafficking domain (MITD) tag are fused to the coding sequence to augment human leukocyte antigen (HLA) class I and II presentation of the encoded antigen and recognition by CD8^+^ and CD4^+^ T cells^10^. Formulation in liposomal nanoparticles (LPXs) for intravenous administration targets resident dendritic cells in all lymphoid compartments of the body^11,12^ (Fig. 1a). The use of uridine mRNA in the RNA–LPX vaccine technology links antigen delivery with co-stimulation via Toll-like receptor (TLR)-mediated, type-I-interferon-driven antiviral immune responses, resulting in profound expansion of antigen-specific T cells in humans, which is effective even against non-mutant self-antigens^11–13^.

The neoantigen vaccine was evaluated in one of the arms of the exploratory first-in-human TNBC-MERIT (Mutanome Engineered RNA Immuno-Therapy) umbrella study^14^ (ClinicalTrials.gov: NCT02316457), which explored the feasibility, safety and ability to induce antigen-specific immune responses of various types of mRNA cancer vaccines in a non-comparative manner in patients with early-stage TNBC. Patients were eligible within one year of completing standard therapy. Tumour tissue was used for mutation detection and customized vaccine design. Patients received eight doses (6× weekly, 2× biweekly, last dose at day 64) of their personalized neoantigen vaccine composed of a maximum of 20 individual cancer mutations encoded by 2 RNA–LPX molecules (Fig. 1a). Fourteen out of fifteen consented patients received the per protocol treatment and were evaluable (Fig. 1b and Extended Data Table 1). The first three patients started with a 14.4-µg dose of RNA and were escalated to the target dose level of 50 µg. These patients also received an off-the-shelf RNA–LPX-based vaccine encoding non-mutated, shared tumour-associated self-antigens (TAAs) as a bridge until the on-demand manufactured personalized vaccine was available. The subsequent 11 patients were vaccinated with the neoantigen vaccine at 50-μg RNA dose without the bridging TAA vaccine (Extended Data Fig. 1).

Five out of fourteen patients were node-positive at initial diagnosis (Fig. 1b), and all patients had surgery with curative intent (Extended Data Table 1). Pathological complete response (pCR) status was available for three out of seven individuals with prior neoadjuvant chemotherapy—one exhibited pCR (P14) and two exhibited non-pCRs (P3 and P9). The interval between initial diagnosis and enrolment in this trial ranged from 0.56 to 1.53 years. On-demand manufacturing was demonstrated to be feasible in a standard clinical setting, and all intention-to-treat patients received their individualized vaccine. The average turnaround time for vaccine production—defined as the period from sample receipt to vaccine release—was 69 days (range: 34–125 days). Notably, the primary aim was not to optimize turnaround time, but rather to robustly establish an end-to-end manufacturing workflow from surgery to vaccination using this technological platform.

The most frequently reported treatment-emergent adverse events (TEAEs) related to the study drug were typical reactogenicity symptoms such as pyrexia, headache, chills, nausea and fatigue (Extended Data Table 2), which appeared within the first 1–3 days and were mostly of grade 1 or 2 severity. TEAEs were transient in nature, manageable with antipyretics and typically resolved within a day, as previously reported for other cancer vaccines based on this RNA–LPX platform^6,11,15^.

Neoantigen-specific T cell responses in blood samples obtained at baseline and 7–14 days following the eighth (final) vaccine dose were assessed by two complementary IFNγ enzyme-linked immunospot (ELISpot) assay approaches. For all 14 individuals, we performed ex vivo ELISpot, which detects high-magnitude, late-differentiated T cell responses following overnight incubation of peripheral blood mononuclear cells (PBMCs) with overlapping peptides (OLPs) spanning the sequence of vaccine-encoded neoantigens for each individual. Eight patients had sufficient PBMCs left for additional testing by ELISpot after in vitro stimulation (IVS) of CD4^+^ and CD8^+^ T cells for 1.5 weeks with autologous antigen-presenting cells loaded with OLPs spanning vaccine-encoded neoantigens. This method captures low-frequency, early-differentiated T cells with high proliferative potential, which facilitates detection of CD4^+^ T cell responses (which tend to be of low frequency).

**Fig. 2:**
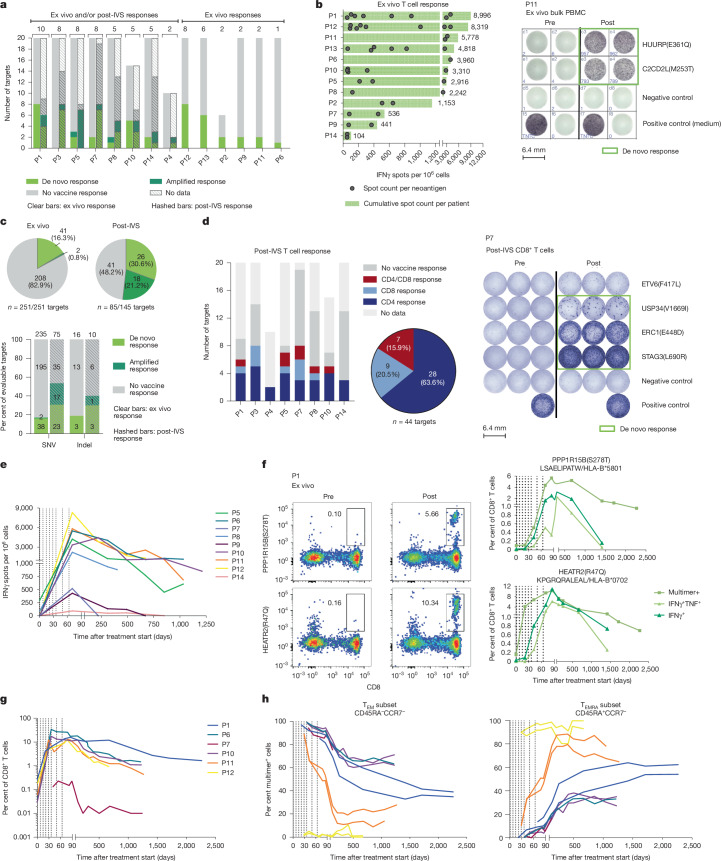
Neoantigen vaccine-specific T cells were induced in all patients and were mostly high-magnitude, durable, poly-epitopic and polyfunctional, and of T_eff_ or T_EM_ phenotype. **a**, Number of neoantigen vaccine-induced T cell responses in patients (P1−P14) measured in PBMCs by IFNγ ELISpot ex vivo (*n* = 14) and after IVS (*n* = 8). Total number of immunogenic mutations is shown above the bars. **b**, Left, magnitude of T cell responses to individual neoantigens detected by ex vivo IFNγ ELISpot for all participants. Right, ELISpot data for P11. TNTC, too numerous to count. **c**, IFNγ ELISpot T cell responses. Top, ex vivo (left; 251 vaccine-encoded mutations across 14 patients) and post-IVS (right; 85 out of 115 vaccine-encoded mutations across 8 patients, owing to limited sample availability). Bottom, comparison of ex vivo and IVS responses for SNVs (clear bars) and indels (dashed bars) as percentage of evaluable targets. **d**, T cell response type by IFNγ ELISpot post-IVS at individual (left) and cohort (middle, showing positive responses) levels. Right, ELISpot data for P7. Data in **a**–**d** are T cell responses detected 7–14 days after final vaccination. **e**, Magnitude of vaccine-induced multi-neoantigen responses per patient over time, showing sum of spots measured in ex vivo INFγ^+^ ELISpot for each of their immunogenic vaccine neoantigens (range: 2–8). For P14, only one out of two neoantigens with a vaccine-included response could be tested. **f**, Characterization of two of the ten neoantigen-specific T cell responses of P1 by HLA-multimer staining. Left, CD8^+^ T cells directed against 2 neoantigen-specific vaccine antigens. Right, intracellular cytokine staining of multimer-positive neoantigen-specific CD8^+^ T cells in PBMCs stimulated with peptides representing the respective neoantigens. **g**,**h**, Cumulative frequency (**g**) and phenotype (**h**) of vaccine-induced, neoantigen-specific CD8^+^ T cells by multimer staining (ten neoantigens, six patients). Curves represent the sum of multimer data for two neoantigens for P1, P10, P11 and P12; P6 and P7 only showed responses to one antigen. In **e**–**h**, dotted lines indicate vaccinations.

**Extended Data Fig. 2:**
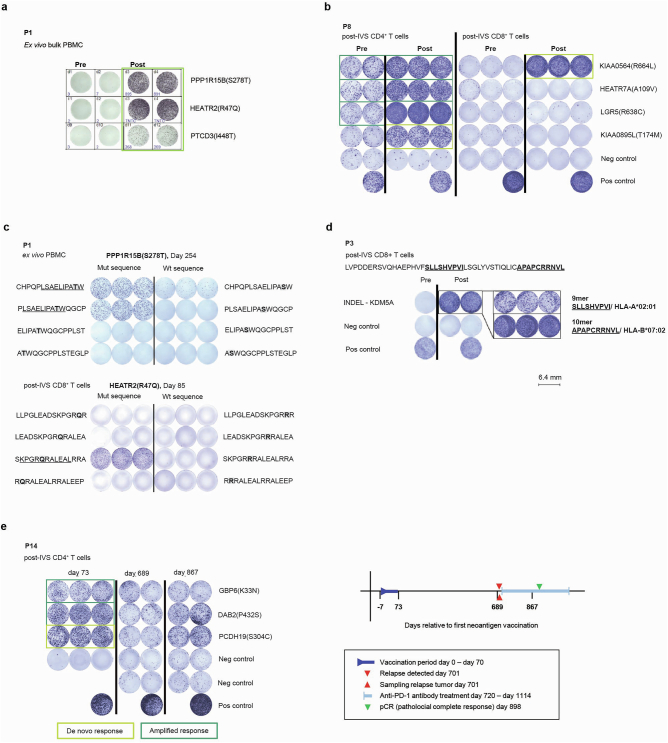
Characterization of vaccine-induced T-cell responses in selected patients. (**a**) Three of eight vaccine-induced de novo T-cell responses detected in patient P1 by bulk PBMC ex vivo ELISpot. (**b**) Concomitant vaccine-amplified CD4+ and de novo CD8 + T-cell response against KIAA0564(R664L) and CD4+ responses (de novo induced [lime green box] and vaccine-amplified [dark green box]) against 3 other neoantigens determined by post-IVS IFNγ-ELISpot in P8. (**c**) Neoantigen-specific CD8 + T-cell responses detected by bulk PBMC ex vivo ELISpot for PPP1R15B(S278T) (upper panel) and after post-IVS IFNγ ELISpot for HEATR2(R47Q) (lower panel) in P1. Individual overlapping peptides encoding the mutated (left) or the wildtype sequence (right) were used (mutated amino acids are shown in bold and highlighted in gray, recognized minimal epitopes are underlined). (**d**) De novo vaccine-induced CD8 + T cells against an KDM5A INDEL recognizing two different epitopes presented by two different HLA restriction elements detected by post-IVS IFNγ ELISpot in P3. Minimal epitopes in bold and underlined. HLA restriction was identified using HLA-multimer staining (data not shown). (**e**) Detection of CD4 + T-cell responses identified as de novo (light green box) or amplified (dark green box) at the end of treatment (d73), day 689 (prior to relapse), and day 867 (during anti-PD-1 antibody treatment), with the timing of sample collection in relation to the vaccination period, time of relapse, sampling of relapse tumor, anti-PD-1 antibody treatment and the diagnosis of pathological complete response. (**a**-**c**, **e**) ELISpot read-out was performed against a pool of overlapping peptides covering the length of each neoantigen, and (**c**) against minimal epitopes for further fine-dissection. Neg control = negative control using dendritic cells loaded with irrelevant peptides, pos control = positive control anti-CD3.

All 14 patients mounted vaccine-induced or amplified T cell responses against one to ten of their individual vaccine targets, as detected by at least one ELISpot method (Fig. 2a). In all but one individual, responses were directed against multiple vaccine antigens. Nine patients had responses to at least five neoantigens (exemplified by patient P12 with eight vaccine neoantigen-directed de novo T cell responses in Fig. 2a). In 12 patients (86%), responses against individual vaccine neoantigens were measurable by ex vivo ELISpot. Eight of these twelve individuals demonstrated responses to single neoantigens reaching 2,000–4,000 IFNγ^+^ spots per 10^6^ PBMCs (Fig. 2a,b and Extended Data Fig. 2a). T cells against 41 of the 251 mutations used across the 14 patients were not detectable prior to vaccination and became detectable after vaccination. T cells against two neoantigens were present at baseline and were further expanded by vaccination (Fig. 2c, top left and Supplementary Data 1). Four individuals exhibited de novo responses against known cancer driver genes (*CREBBP*, *DAXX*, *IGF1R* or *TP53*) (Supplementary Data 1, second tab).

Post-IVS ELISpot analysis of 85 mutations used for vaccination across 8 patients identified specific T cells against 44 (51.8%) of these mutations. More than half (*n* = 26) were induced de novo (Fig. 2c, top right). Around two-thirds (28 out of 44, 64%) of the neoepitopes that were immunogenic in post-IVS ELISpot were recognized by CD4^+^ T cells, 20% (9 out of 44) were recognized by CD8^+^ cytotoxic lymphocytes (CTLs) (Fig. 2d) and 16% (7 out of 44) were concomitantly recognized by CD4^+^ and CD8^+^ T cells, as illustrated by patient P8, who had a pre-existing vaccine-amplified CD4^+^ T cell response and a de novo induced CD8^+^ T cell response against the neoantigen KIAA0564(R664L) (Extended Data Fig. 2b).

Mutation specificity of vaccine-induced T cells was shown for one patient (P1) (Extended Data Fig. 2c), and has been demonstrated with this neoantigen mRNA vaccine technology previously^4^. Single nucleotide variants (SNVs) as well as insertions/deletion mutations (indels) induced T cells; indels were able to provide multiple epitopes, as exemplified by patient P3, who showed T cell responses against independent epitopes presented on different MHC class I alleles derived from a frameshift deletion in *KDM5A* (Extended Data Fig. 2d and Supplementary Table 1).

**Extended Data Fig. 3:**
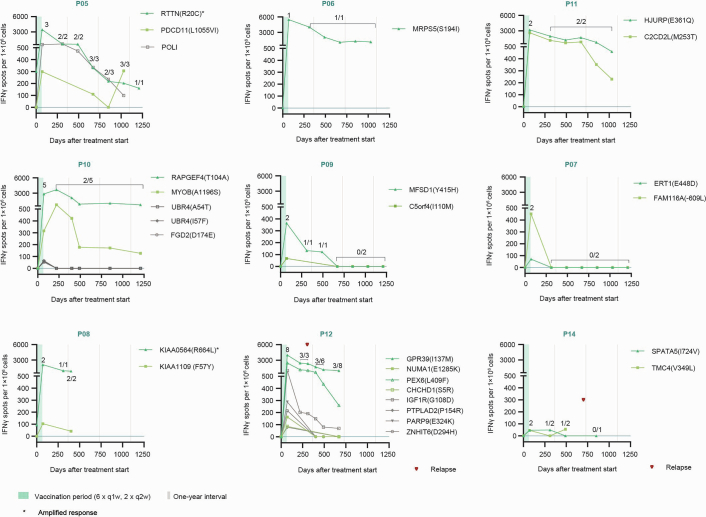
Long-term follow-up of neoantigen-induced ex vivo T-cell responses in evaluable patients. T-cell responses against vaccine-encoded neoantigens measured by ex vivo INFγ ELISpot in bulk PBMCs of single patients (post-IVS data not included). Each symbol represents the normalized mean spot count from duplicate wells for one neoantigen after subtraction of the medium-only control. Ratios above the data points are the number of neoantigens with detectable immune responses within the total number of analyzed neoantigens analyzed at each time point.

Nine patients, with a total of 27 immunogenic mutations, were evaluable for the durability of vaccine-induced immune responses by ex vivo ELISpot, as they had provided blood samples during long-term follow-up. The vaccine-induced multi-neoantigen immune response (sum of immune response magnitude for each of the individual vaccine neoantigens) of patients P5, P8, P6, P10, P11 and P12 underwent a steep expansion followed by a short contraction, and then remained at high levels over a period of 1 to 3.5 years (Fig. 2e and Extended Data Fig. 3). In patients P7 and P9, the expansion and persistence of neoantigen-specific T cells was modest and the response in P14 was very weak. On the level of individual neoantigen-specific T cell responses, in all but one individual (P7), at least one of their vaccine-induced T cells remained detectable for at least 12 months without vaccine boosters (Fig. 2e and Extended Data Fig. 3).

For six of the patients with detectable ex vivo responses, we were able to generate functional peptide–MHC class I complexes for two of their vaccine-induced T cell specificities (only one for P6 and P7) and could confirm the kinetics of CD8^+^ T cell responses by multimer staining. As exemplified for P1 on the individual neoantigen level (Fig. 2f; gating strategy in Extended Data Fig. 7a) and shown for all six patients for their cumulative multiple-neoantigen response (Fig. 2g), de novo induced mutation-specific CD8^+^ T cells expanded rapidly during vaccination, became detectable after 3 vaccine doses (around week 3) and reached a median frequency of 6.58% (range: 0.5–17.5%) among all circulating CD8^+^ T cells after the final vaccination. Several of the neoantigen-specific CD8^+^ T cell specificities remained detectable in the blood at single-digit percentages 1 to 6 years after the final vaccination (Fig. 2g). For example, at the end of the vaccination regimen, 10.3% of circulating CD8^+^ T cells in P1 recognized HEATR2(R47Q), one of the 10 mutations against which the patient had developed a de novo immune response. Two years later, despite the absence of vaccine boosters, these cells (representing only a fraction of the vaccine-induced polyneoantigenic T cell response of P1) still represented more than 3% of the circulating CD8^+^ T cells (Fig. 2f). P6 had 17.5% de novo induced T cells against MRPS5 (S194I) after vaccination, which declined to 1.5% over a period of 3 years (Fig. 2g). The majority of the vaccine-induced CD8^+^ T cells were of effector memory phenotype in the first months and developed towards a more late-differentiated CD45-re-expressing phenotype over 3 to 6 years (Fig. 2h), except in P12, in whom almost all neoantigen-specific T cells were of the late-differentiated phenotype after 4 vaccinations (Fig. 2h).

Owing to the long-term remission of P1 (who remained relapse-free for more than six years following vaccination) and the availability of PBMCs collected throughout this period, this individual served as an informative case for detailed analysis of vaccine-induced T cell responses. P1 generated T cell responses against 10 vaccine-targeted mutations, including strong ex vivo IFNγ-secreting responses to 8 neoantigens, amounting to approximately 9,000 spot-forming units per million PBMCs (Fig. 2b, left and Extended Data Fig. 2a). Additional responses to two neoantigens were detected at lower magnitude post-IVS and were mediated by CD4^+^ T cells (Fig. 2a and Supplementary Table 1).

**Fig. 3:**
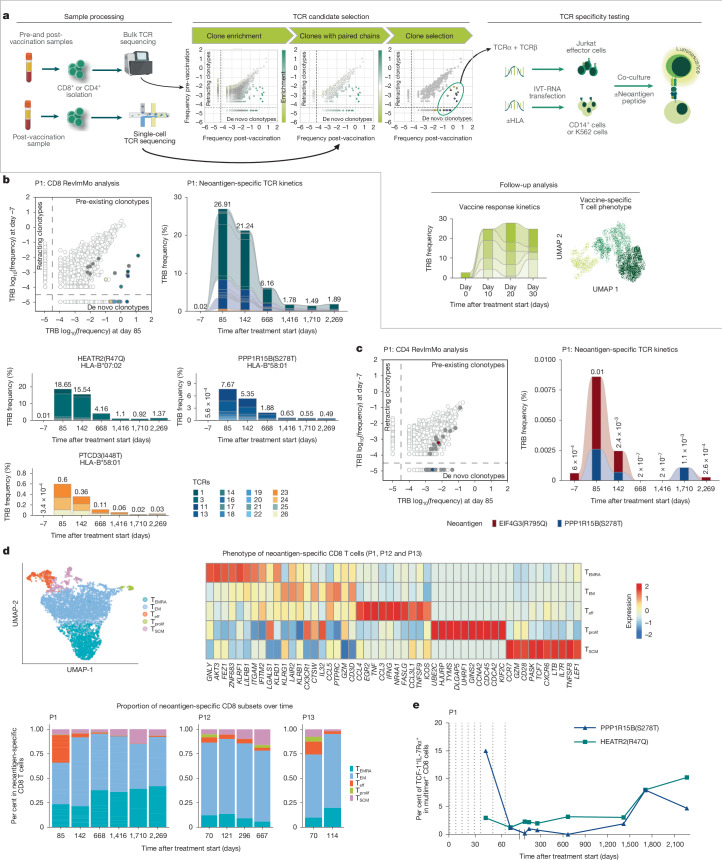
Neoantigen vaccine-induced long-lasting cytotoxic and self-renewing neoantigen-specific T cells in patient P1. **a**, RevImMo approach for identification and functional characterization of vaccine-expanded neoantigen-specific T cell clones. Bulk TCR sequencing data are generated from pre- and post-vaccination CD8^+^ or CD4^+^ T cells. Post-treatment enrichment is calculated for each TCRβ chain (TRB) CDR3 clonotype. Single-cell TCR sequencing data from post-treatment CD8^+^ or CD4^+^ T cells are used to retrieve the relative paired TCRα chain (TRA) and TRB. Only clonotypes with paired chain data are considered in the enrichment analysis, and up to 30 of the most highly enriched are selected for TCR cloning and in vitro specificity testing. Jurkat effector cells are transfected with TCR chain-encoding RNAs and co-cultured with antigen-presenting cells transfected with the individual’s HLA class I or II molecules and loaded with peptides representing the neoantigen targets of their vaccine. TCR reactivity is determined by assessing TCR signalling via NFAT-induced luciferase reporter activity. UMAP, uniform manifold approximation and projection. Created in BioRender. Cortini, A. (2025) https://BioRender.com/6smlkd5. **b**,**c**, RevImMo analysis and TCR kinetics for P1. Top left, bulk TCR profiling data of CD8^+^ and CD4^+^ T cell clones in blood pre- and post-vaccination. RevImMo-selected clones are highlighted; vaccine neoantigen-specific TCRs are presented as filled circles, TCRs with unresolved specificity are shown as unfilled circles. Tracking of neoantigen-specific clones in bulk CD8^+^ (**b**, top right) and CD4^+^ (**c**, right) TRB profiling data from longitudinal blood samples. **b**, Bottom, frequency of all TCRs and separation by specificity. **d**, Unsupervised clustering analysis of neoantigen-specific CD8^+^ T cells from longitudinal blood samples of P1, P12 and P13 (top left) and top 10 differentially expressed genes in each neoantigen-specific cell cluster (top right). Bottom, proportion of neoantigen-specific cell subsets in the longitudinal blood samples. **e**, Frequency of TCF-1^+^IL-7Rα^+^ in the neoantigen-specific CD8^+^ T cell population of patient P1 identified via multimer staining. Dotted lines indicate vaccinations.

**Extended Data Fig. 4:**
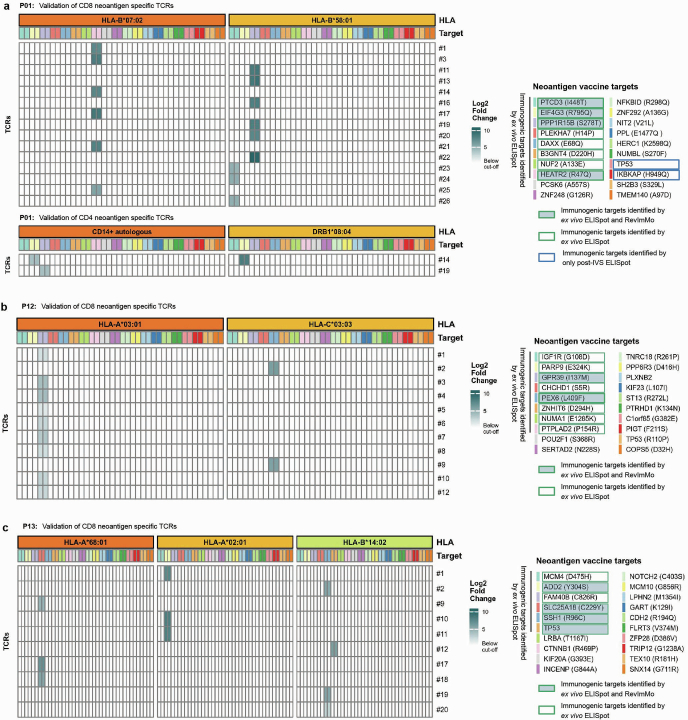
In vitro validation of vaccine-induced neoantigen-specific CD8+ and CD4 + TCRs identified in patients P01, P12 and P13. Jurkat-NFAT reporter assay analysis of neoantigen-specific TCRs cloned from P01 (**a**), P12 (**b**) and P13 (**c**) in conjunction with the patient’s respective restriction elements using overlapping peptide pools (15mers) encoding for the single vaccine antigens. Fold change of the NFAT-induced luciferase signal after co-culture with target cells compared to TCR-transfected effectors only are shown for each TCR. The neoantigen specificity of P01’s CD8 TCR #18 was determined by multimer staining.

To dissect the T cell repertoire of P1, we used reverse immunomonitoring (RevImMo), which combines bulk and single-cell T cell receptor (TCR) sequencing to identify neoantigen-specific clonotypes expanded following vaccination. Twenty-six clones enriched in the post-vaccination single-cell TCR-sequencing data were cloned and tested using an enhanced Jurkat-NFAT reporter assay^6^ (Fig. 3a). Of the 26 most enriched CD8^+^ T cell clones with paired TCR sequences, 16 recognized the three ELISpot-positive vaccine targets—HEATR2(R47Q), PPP1R15B(S278T) and PTCD3(I448T)—presented by major histocompatibility complex class IB (HLA-B) alleles (Fig. 3b and Extended Data Figs. 2a and 4a). Eleven of these TCRs were de novo induced; the remaining five were pre-existing.

Tracking via CDR3 sequences revealed that neoantigen-specific CD8^+^ T cells persisted at approximately 1.9% of the CD8^+^ TCR repertoire over 6 years post-vaccination in P1 (Fig. 3b), consistent with multimer analysis results for PPP1R15B(S278T) and HEATR2(R47Q) (Fig. 2f, right). Using intracellular cytokine staining, we showed that these two durable T cell populations produced TNF and IFNγ for up to 3.5 years following the patient’s last vaccination (Fig. 2f, right and Extended Data Fig. 7b,c). Additionally, two low-abundance CD4^+^ TCRs recognizing separate vaccine targets were detected up to 4.5 and 6 years post-vaccination, respectively (Fig. 3c).

**Extended Data Fig. 5:**
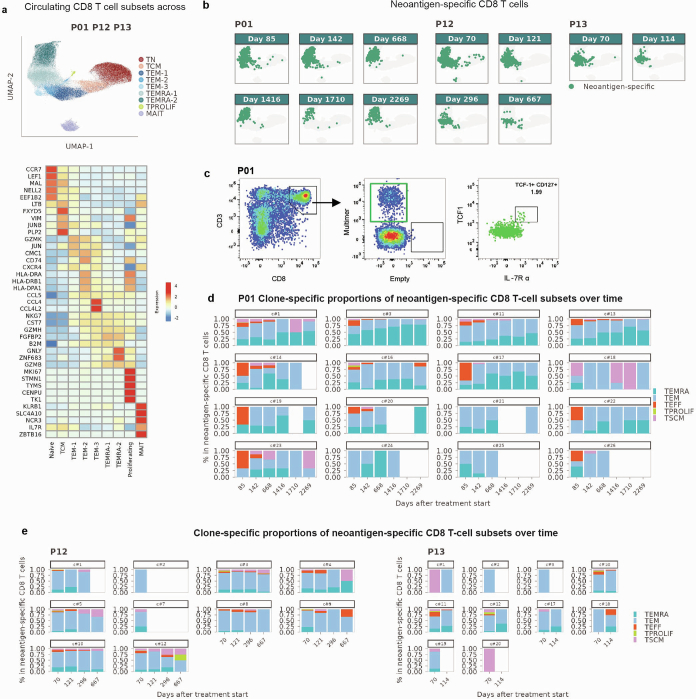
Phenotype of neoantigen vaccine-induced CD8 + T-cell clones in P01, P12 and P13. (**a**) Cell subsets identified by unsupervised clustering analysis of blood CD8 + T cells (upper panel), and the top 5 differentially expressed genes in each cluster (lower panel). (**b**) Distribution of neoantigen-specific T cells in circulating CD8 + T-cell subsets in longitudinal blood samples collected from P01, P12 and P13. (**c**) Gating strategy (multimer-positive cells in green) for measuring frequency of TCF-1 + CD127+ in the neoantigen-specific CD8 + T-cell population as identified via multimer staining (data shown in Fig. 3e). (**d**, **e**) Proportion of neoantigen-specific cell subsets in longitudinal blood samples collected from P01 (D), P12 and P13 (**e**) shown per clone. TEMR, terminally differentiated effector T cells; TEM, effector memory T cells; TEFF, activated effector memory T cells; TPROLIF, proliferating T cells; TSCM, stem-like memory phenotype.

We expanded TCR repertoire-level analysis to include patients P12 and P13, tracking neoantigen-specific CD8^+^ T cells using TCR sequences identified in longitudinal single-cell RNA-sequencing (RNA-seq) and TCR-sequencing datasets (Extended Data Fig. 5a,b). Unsupervised clustering of the total CD8^+^ T cell population revealed nine transcriptional subsets indicative of naive T cells (TN), central memory T cells (T_CM_), three subsets of effector memory T cells (T_EM_1 to T_EM_3), two terminally differentiated effector T cell subsets (T_EMRA_1 and T_EMRA_2), mucosal-associated invariant T (MAIT) cells, and a cluster of proliferating T cells (Extended Data Fig. 5a). Within these subsets, 1 to 2 weeks after the final vaccine dose, neoantigen-specific CD8^+^ T cells predominantly exhibited a terminal effector memory re-expressing CD45RA (T_EMRA_) phenotype, which persisted as the dominant subset throughout follow-up (Extended Data Fig. 5b).

We identified five transcriptionally distinct clusters within the neoantigen-specific CD8^+^ T cell pool: a T_EMRA_ cluster marked by *GNLY*, *ZNF683* and other genes associated with cytotoxicity and tissue residence; a T_EM_ cluster expressing *KLRG1*, *LAIR2* and *CCL5*, consistent with a memory-like profile; an activated effector memory T cells (T_eff_) group producing cytokines (*CCL4*, *TNF* and *IFNG*) indicative of active effector function; a proliferating T (T_prolif_) cell group characterized by cell cycle genes (*UBE2C* and *KIF2C*); and a stem-like memory phenotype (T_SCM_) subset co-expressing *CCR7*, *TCF7*, *LEF1*, *IL7R* and other markers of stem-like memory and naive lineage (Fig. 3d, top). Early responses at the end of treatment (day 70–85) were dominated by T_EM_ and T_eff_ cells (Fig. 3d, bottom and Extended Data Fig. 5d,e). Over time, the T_eff_ cells were progressively replaced by T_EMRA_ cells, which together with T_EM_ cells became the predominant phenotypes during long-term follow-up. Notably, stem-like memory T_SCM_ populations also emerged and persisted, supporting the presence of durable and phenotypically diverse memory T cell pools (Fig. 3d, bottom Extended Data Fig. 5d,e). Using flow cytometry in P1, we confirmed persistence of stem-like vaccine-induced neoantigen-specific T cells co-expressing *TCF-1* (also known as *TCF7*) and *IL7RA* (also known as *IL7R*) for more than 6 years following vaccination (Fig. 3e and Extended Data Fig. 5c), highlighting the persistence of a memory T cell pool with regenerative potential.

**Extended Data Fig. 6:**
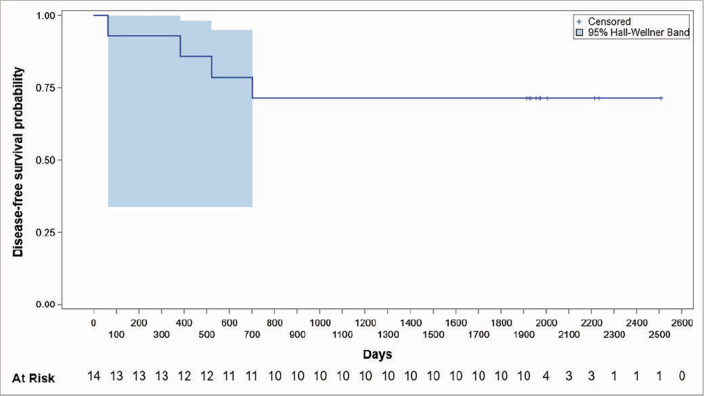
Kaplan–Meier curve time-to-event analysis for disease-free survival (DFS) probability. Disease-free survival was defined for this non-protocol specified analysis as the time from first vaccine dose to cancer recurrence or death from any cause. Imaging after initial (neo)adjuvant treatment was only performed upon suspected progression, which may overestimate DFS. Date of last visit/contact served as the date of censoring (labeled as +).

As of February 2025, out of 14 patients, 10 remained relapse-free with a median follow-up of 5 years (median 62 months, range 15–80 months) after the last vaccine dose (Fig. 1b and Extended Data Fig. 6); in addition, patient P8 remained relapse-free until their death from unknown causes 15 months after the last vaccine dose. Patients P12, P13 and P14 experienced disease recurrence. P14 had the weakest vaccine-induced immune response of the treated patients. Ex vivo ELISpot data showed that T cells against two vaccine neoantigens were present with abundances an order of magnitude lower than those observed in most other patients shortly after the last vaccine dose, but not at later time points, and post-IVS captured vaccine-amplified CD4^+^ T cells against three additional vaccine neoantigens (Fig. 2b left and Extended Data Fig. 3). Twenty months after the last vaccine dose, P14 had locoregional recurrence of disease in the right breast and axilla. The new lesion was infiltrated with T cells, and tumour cells displayed robust MHC class I expression (Extended Data Fig. 8a). This individual was treated with anti-PD-1 antibody and sequential chemotherapy and experienced a complete response, which lasted for 15 months, after which there was a systemic relapse and the patient died three months later. Three vaccine-induced CD4^+^ responses identified in this patient one week after the last vaccine dose remained detectable up to day 867, which was subsequent to the recurrence and during anti-PD-1 treatment (Extended Data Fig. 2e). The other two patients with tumour recurrences (P12 and P13) raised high-magnitude, vaccine-induced responses against multiple vaccine targets (Fig. 2b, left).

**Fig. 4:**
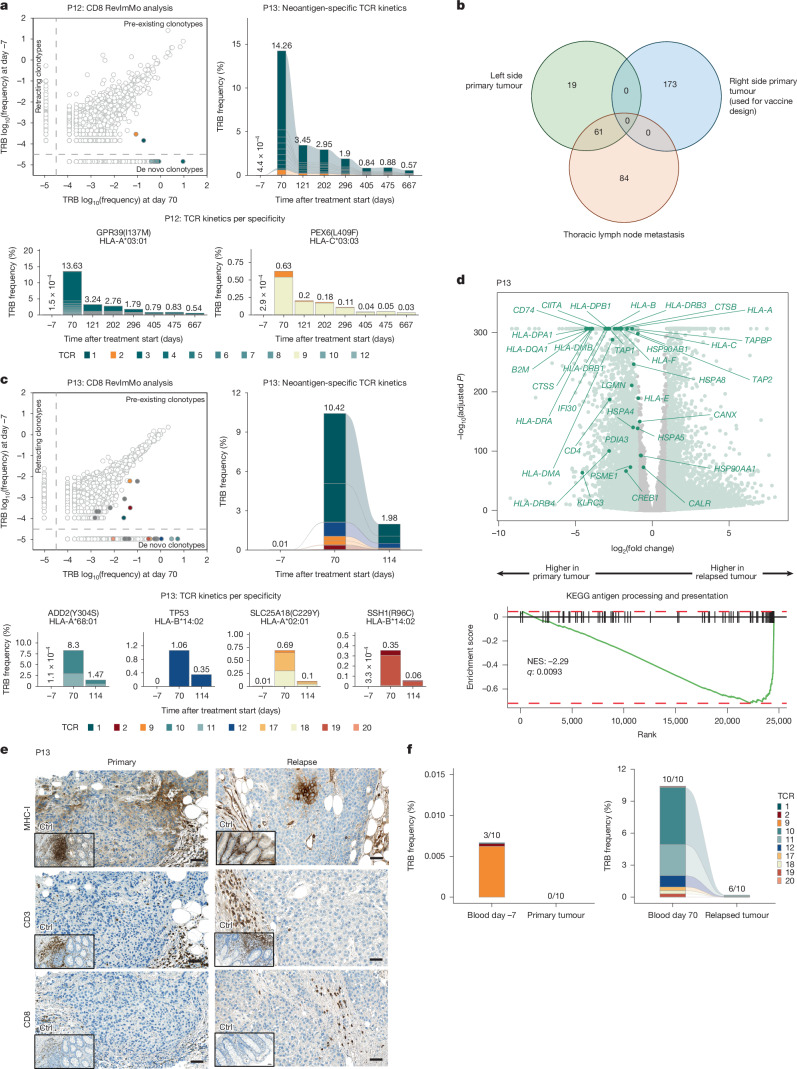
T cell response and tumour characteristics in two patients with tumour recurrence. **a**,**c**, Characterization of neoantigen-specific T cell clones in P12 (**a**) and P13 (**c**). Vaccine-induced expansion of neoantigen-specific T cell clones was assessed by RevImMo analysis comparing pre- and post-vaccination blood samples. Top left, bulk TCR profiling scatter plots; selected clones with neoantigen-specific TCRs (filled circles) and with TCRs of unresolved specificity (unfilled circles). Bar graphs track neoantigen-specific clones in bulk CD8^+^ TRB profiling data from longitudinal blood samples, showing cumulative frequency of all TCRs (top right) and separated by specificity (bottom). **b**, Comparison of SNVs called in the different tumour tissue samples obtained from patient P12. **d**, Top, differentially expressed genes between pre-treatment and relapse tumour tissue. Light green, genes with |log_2_(fold change)| > 1 and *q*-value < 0.01 (Wald test, two-sided, adjusted with Benjamini–Hochberg procedure); dark green, leading edge genes from gene set enrichment analysis (GSEA) of Kyoto Encyclopedia of Genes and Genomes (KEGG) ‘antigen processing and presentation’ pathway (bottom); NES, normalized enrichment score. **e**, Staining of post-vaccination formalin-fixed paraffin-embedded (FFPE) sections of relapsed tumour tissue from P13 and of the pre-vaccination tumour (single staining). Insets show staining of colon tissue as positive control. Scale bars, 50 μm. **f**, Cumulative frequency of neoantigen-specific TCRs in CD8^+^ T cells in blood and tumour of P13. Shared clonotypes among pre-vaccination blood and primary tumour (left) and post-vaccination blood and relapsed tumour (right). The TRB CDR3 sequences were used for tracking in bulk profiling data. Numbers above bars: number of neoantigen-specific TCRs detected versus the total identified.

Patient P12, who had *BRCA1*-mutation positive bilateral TNBC, underwent surgery for two tumours, T1cN0 in the right breast and T1cN1 in the left breast, followed by adjuvant chemotherapy and personalized neoantigen vaccination. The right breast tumour was sequenced to guide vaccine design. P12 developed a robust neoantigen-specific T cell response against eight targets, as confirmed by ex vivo ELISpot (Fig. 2b, left and Supplementary Data 1). Through RevImMo (Fig. 4a and Extended Data Fig. 4b) and single-cell TCR sequencing of multimer-enriched populations (Extended Data Fig. 8b), we identified 107 neoantigen-specific TCRs spanning 4 distinct antigen–HLA pairs: GPR39(I173M)–HLA-A03:01, IGF1R(G108D)–HLA-B15:01 and PEX6(L409F) with both HLA-B15:01 and HLA-C03:03. None of these TCRs were present before vaccination, consistent with a strong, polyclonal de novo immune response. Phenotypic profiling revealed a dominant T_EM_ cell response, with progressive differentiation into memory stem-like T_SCM_ cells over time (Fig. 3d, bottom and Extended Data Fig. 5b). T_EMRA_ cells targeting one of the immunodominant neoantigens were first detected after two to three vaccine cycles and persisted long-term (Fig. 2h). Although clonal breadth was maintained, a significant reduction in TCR frequencies was observed during the two months following the final vaccination (Fig. 4a, Extended Data Fig. 8b). Relapse occurred ten months later, initially presenting as *BRCA1*-positive thoracic lymph node metastases and later spreading to the lungs and brain. This patient died from disease progression 17 months post-vaccination. Notably, the metastatic lesion retained robust MHC expression (Extended Data Fig. 8c), but none of the somatic SNVs from the sequenced right breast tumour were present (Extended Data Fig. 8d). Instead, 61 SNVs overlapped with the left breast tumour, indicating that recurrence probably originated from this independently evolved tumour (Extended Data Fig. 4b). In line with this origin, no neoantigen-specific TCRs were detectable in the relapsed metastasis (Extended Data Fig. 8e).

Patient P13 experienced a locoregional recurrence in the right breast soon after completing vaccination and died 13 months later. RevImMo analysis revealed ten CD8^+^ T cell clones specific for four of the six vaccine neoantigens identified by ex vivo ELISpot—ADD2(Y304S), SLC25A18(C229Y), SSH1(R96C)—and a *TP53* indel mutation (Figs. 2b and 4c, Extended Data Fig. 8f, Supplementary Table 2 and Supplementary Data 1). These vaccine-induced T cells resembled those from patient P1 in phenotype but showed a greater enrichment for T_EM_ cell subsets (Fig. 3d and Extended Data Fig. 5b). The relapsed tumour displayed increased variant allele frequencies for most neoantigens included in the vaccine, including those driving strong T cell responses (Extended Data Fig. 8f,g). However, transcriptomic analysis revealed downregulation of key genes involved in antigen presentation—including MHC class I and *B2M*—compared to the primary tumour (Fig. 4d and Supplementary Data 2 and 3). Immunohistochemical staining showed that whereas the primary tumour had low MHC class I expression and minimal T cell infiltration, the recurrent tumour had nearly complete loss of MHC class I and further reduced immune infiltration (Fig. 4e). TCR profiling across blood and tumour tissue indicated that three neoantigen-specific TCRs were present at low frequency in the blood prior to vaccination, but none were detectable in the primary tumour. Following vaccination, ten neoantigen-specific TCRs were identified, with six of these clonotypes present in the relapsed tumour (Fig. 4f). Despite infiltration by functional, vaccine-induced T cells, the recurrent tumour may have escaped immune pressure through downregulation of antigen presentation pathways.

## Discussion

Our study shows the clinical feasibility, safety, and robust immunogenicity of an individualized uridine mRNA-based neoantigen vaccine in patients with TNBC following adjuvant or neoadjuvant therapy. The vaccine was well tolerated and consistently elicited strong neoantigen-specific T cell responses in nearly all patients against several of their neoantigens. Poly-epitopic immune responses in individual patients reached low double digits of T cells in peripheral blood, magnitudes typically observed with adoptive T cell therapies.

We observed several cases with the same mutated sequence giving rise to neoepitopes recognized concomitantly by both CD4^+^ and CD8^+^ T cells, or neoepitopes presented on different HLA class I restriction elements to distinct CD8^+^ T cell clones. Neoantigen-specific T cells remained detectable in a functional state in peripheral blood for years after vaccination, underscoring the potential for lasting immunity. Of note, these T cells not only persisted but underwent further phenotypical evolution. A significant proportion of these T cells matured into late-differentiated cytotoxic effector cells expressing GLNY and ZNF683, indicators of ready-to-act cells associated with favourable clinical outcomes^16–18^.

Simultaneously, a subset of early-differentiated T cells retained high levels of stem cell-like memory markers such as IL-7Rα and TCF7 (encoded by *TCF-1*), a transcription factor that is required for the self-renewal of stem-like CD8^+^ T cells generated in response to viral or tumour antigens, and for preserving heightened responses to checkpoint blockade immunotherapy^19^.

We observed frequent induction of CD4^+^ T cells by the cancer mutations selected for vaccine design. However, vaccine-induced CD4^+^ T cell responses exhibited markedly lower clonal frequencies than CD8^+^ T cells, and owing to limited amounts of PBMCs and technical constraints, we focused our study on CD8^+^ T cells.

Findings in patients P12, P13 and P14 point to different possible mechanisms of treatment failure and escape to neoantigen vaccines. One such mechanism may be the lack of a proficient vaccine-induced immune response in the first place, as observed in patient P14, who stands out in this regard. Although this individual developed a complete response upon anti-PD-1 treatment, an outcome rarely observed in patients with TNBC, the patient subsequently died from a systemic relapse. P12 and P13 experienced tumour recurrence despite having mounted strong multi-neoantigen immune responses.

Patient P13 had very low MHC expression in the initial tumour and loss of MHC class I in the recurrence, most probably driven by downregulation of *B2M*. Complete loss of HLA class I presentation remains an effective escape mechanism, and has been observed in several types of potent T cell based therapies^4,20–23^, highlighting the need for combination therapies such as tumour-targeting antibodies to inhibit the outgrowth of HLA-deficient cancer cells or immunomodulatory approaches to restore neoantigen recognition in β2M-deficient tumours^24,25^.

The third patient with relapse, P12, had BRCA-mutated TNBC, which is associated with independently evolving unrelated synchronous or metachronous primary tumours^26–28^. The bilateral breast tumours in patient P12, only one of which was used to inform vaccine design, were found to be clonally independent at the time of diagnosis. Sequencing of multiple lesions may be advisable in genetic predispositions with the risk of antigenically distinct primaries.

Limitations of the study include the small number of participants, particularly in the cohort in which deeper immunologic analyses were conducted, and the absence of control groups to further investigate possible immune escape.

The TNBC study extends our previous findings from other tumour types and shows that this personalized vaccine approach can be broadly used. In melanoma, we demonstrated a profound reduction in the cumulative rate of metastatic events, indicating the applicability of mRNA neoantigen vaccine to tumours with high mutation load^4^. In pancreatic cancer, a tumour with low number of mutations and highly immune suppressive environment, this vaccine induced immune responses in 50% of patients, which correlated positively with clinical outcome^5^. In TNBC, a cancer with low to moderate mutational load, we now demonstrate that all patients developed neoantigen-specific T cell responses with more than 85% being of high magnitude. TNBC has a poor prognosis, especially in patients who do not achieve pathological complete response after completion of neoadjuvant chemotherapy^29–31^. Strong immunogenicity of the neoantigen vaccine in the majority of patients, durability of the induced T cell responses, and early signs of clinical activity warrant further clinical testing in this patient population.

## Methods

### Trial design and data reporting

The primary objective of this open-label, first-in-human, phase 1, three-arm umbrella trial (ClinicalTrials.gov: NCT02316457) was to separately assess the feasibility, safety, and tolerability profile of two different mRNA–LPX-based vaccine types: an off-the-shelf warehouse vaccine composed of non-mutated TAAs and an on-demand manufactured individualized neoantigen vaccine. Vaccine-induced antigen-specific immune responses were investigated (secondary endpoint). Here, we report findings related to the individualized neoantigen vaccine arm of this trial. The trial was carried out in Germany and Sweden in accordance with the Declaration of Helsinki and Good Clinical Practice Guidelines, and with approval by the independent ethics committees (Ethik-Kommission of the Landesärztekammer Rheinland Pfalz, Mainz, Germany and Regionala Etikprövningsnämnden, Uppsala, Sweden) and the competent regulatory authority (Paul-Ehrlich Institute, Langen, Germany and Medical Products Agency, Uppsala, Sweden). All patients provided written informed consent.

Eligibility criteria were: histologically confirmed invasive adenocarcinoma TNBC (pT1cN0M0–[any]T[any]NM0); previous standard of care treatment (that is, neoadjuvant chemotherapy of the primary tumour followed by surgery or surgery and adjuvant chemotherapy); prior radiotherapy was allowed; at least 18 years of age; adequate haematopoietic, hepatic and renal function; tumours expressing at least 5 neoantigens. Patients were eligible for enrolment within one year after completion of standard of care therapy per local policy (for example, surgery and/or chemotherapy and/or radiotherapy). Key exclusion criteria were the recurrence of breast cancer prior to the start of trial treatment and presence of clinically relevant autoimmune disease or active viral infections. The individualized neoantigen vaccine consisted of two single-stranded RNA molecules each encoding up to ten neoantigen vaccine targets selected based on somatic mutation analysis of each patient’s tumour. RNAs were liposomally formulated into RNA–LPX for intravenous administration as described^11^. For patients treated with neoadjuvant chemotherapy, the FFPE tumour sample from the diagnostic core biopsy was used for analysis of tumour antigen expression. In exceptional cases (for example, low sample quality), the resected FFPE tumour tissue from surgery could be used for analysis of antigen expression instead. agCapture 3.4.2.6 (ArisGlobal) was used for electronic data capture.

The clinical trial report of the primary and secondary endpoints assessed in the main study phase until end of treatment and a 56-day follow-up period in 2020 was submitted to health authorities in spring 2021. Beyond this follow-up, the three patients pretreated with bridging TAA vaccine were followed up passively; one patient consented to provide blood samples via a research project. The following 11 patients (only treated with neoantigen vaccine) participated in an active long-term follow-up for 3 years until 2023. The data generated in the long-term follow-up have been summarized in an addendum to the clinical trial report and submitted to health authorities in the spring of 2024. Following this follow-up period, these patients were then followed up passively.

### Next generation sequencing

Tumour DNA was extracted from three 10 μm curls of FFPE tumour tissue in duplicates using a modified version of QIAamp DNA FFPE Tissue kit (Qiagen). RNA extraction was done in duplicates using the ExpressArt Clear FFPE RNAready from AmpTec. For DNA extractions from PBMC cells, the DNeasy Blood and Tissue Kit (Qiagen) was used. Extracted nucleic acids were used for generation of various NGS libraries. Targeted RNA-seq libraries were constructed in duplicate from FFPE tumour tissue RNA using the NEBNext RNA First Strand Synthesis Module and NEBNext Ultra Directional RNA Second Strand Synthesis Module for cDNA syntheses and a modified version of SureSelect XT V6 Human All Exon (Agilent) using 100 ng total RNA input. DNA whole-exome libraries were constructed in duplicates from 100 ng of FFPE tumour DNA and matching PBMC DNA using a modified version of SureSelect XT V6 Human All Exon (Agilent). NGS libraries for whole-exome sequencing of the tumour and matching PBMCs were prepared by fragmenting 100 ng genomic DNA in a total volume of 15 μl using microTUBE-15 AFA Beads Screw-Cap (Covaris) to an average fragment length of approximately 150 bp. For NGS, the libraries were diluted to 3 nM and clustered at 10 pM using the Illumina HiSeq 3000/4000 PE Cluster Kit. Pooled exome library from FFPE and PBMC DNA were sequenced as 4-plexes on three lanes, whereas the RNA library replicates were sequenced as 2-plexes in one lane. All libraries were sequenced paired-end 50 nt on an Illumina HiSeq 4000 platform using two HiSeq 3000/4000 SBS 50 cycles kits (Illumina). For P12, post-vaccination samples were paired-end (100 nt) sequenced on an Illumina NovaSeq 6000 platform using an Illumina NovaSeq 6000 S2 Flow Cell and an Illumina NovaSeq 6000 S2 Reagent Kit v.1.5 (100 Cycles) instead.

### Bioinformatics and mutation discovery

All genomics-related data analysis steps were coordinated by proprietary bioinformatic pipeline (BioNTech) implemented in the Python programming language and described in brief below. For each of the replicate of the DNA libraries, at least 180 × 10^6^ paired-end 50 nt reads were available covering ≥70% of targeted bases with ≥100× coverage. For the RNA libraries a minimum of 75 × 10^6^ paired-end 50 nt reads were required.

For mutation detection, DNA reads were aligned to the reference genome hg19 with bwa (v.0.7.10)^32^. The resulting alignment files were converted to BAM format using SAMtools (v.0.1.19)^33^. Somatic SNVs were called using an in-house mutation caller^4^ and short insertions and deletions were called using Strelka^34^, by comparing the aligned reads of the tumour DNA to those of the matching PBMC DNA.

Genomic coordinates of identified somatic variants were compared with the UCSC Known Genes transcript coordinates to associate the variants with genes, transcripts, and potential amino acid sequence changes. Synonymous and non-sense mutations were filtered. For verified and non-synonymous cancer mutations that were selected as potential neoantigen vaccine targets, a mutated peptide sequence (MPS) was determined based on the mutated transcript sequence for contribution to design of a MPS concatemerized vaccine. In the case of SNVs, these were the 27mer peptide regions with the changed amino acid in the centre. For insertions and deletions resulting in a frameshift, the MPS featured the sequence from the changed amino acid to the next stop codon (maximum 50 amino acids). Germline variants in the region of the mutated peptides were identified using SAMtools based on the matching PBMC DNA. Protein changing germline variants were first phased based on the RNA-seq reads and, if in-phase with the somatic mutations, included in the patient-specific neoantigen vaccine target.

For RNA-seq, RNA reads were aligned to the hg19 transcriptome using sailfish (v.0.7.6)^33^ to estimate the transcript expressions. Non-expressed transcripts were filtered.

Further, RNA reads were aligned to the hg19 reference genome using STAR (v.2.4.2a)^35^ for phasing somatic with germline variants, as well as for determining the relative expression of a mutated transcript in comparison to the transcript not carrying the somatic mutation.

NGSCheckMate (v.1.0)^36^ was used to confirm the identical patient as origin for all DNA and RNA libraries analysed under the same patient ID.

### Neoepitope prioritization and selection

HLA binding affinity was predicted via the IEDB T cell prediction tools (v.2.13)^37^. For HLA class I the affinity of all variant-containing 8–11mers of the MPS for HLA-A/B/C was predicted using the IEDB-recommended mode. For HLA class II, the affinity of all variant-containing 15mers of the MPS for HLA-DRB was predicted using the consensus3 method. Out of all predictions for a single variant, the best consensus score was associated with the respective MPS.

Up to 46 MPS from all identified MPS were prioritized using the same in-house bioinformatics pipeline using the sorting and filters described below. First, only somatic mutations with a variant allele frequency in RNA > 0 were considered. From these: (1) up to 5 MPS from insertions or deletions were selected based on their HLA class I binding affinity; (2) up to 20 MPS from SNVs based on their HLA class II binding affinity and transcript expression ≥10 RPKM; (3) up to 20 MPS from SNVs based on their HLA class I binding affinity and transcript expression ≥1 RPKM; (4) further MPS from SNVs based on their transcript expression to reach 46 MPS in total; in case less than 46 MPS could be selected, somatic mutations with a variant allele frequency in RNA of zero were added based on (6) HLA class I score; and (6) transcript expression.

An algorithm implemented in R was used to select up to 20 neoantigen vaccine targets from the list of prioritized MPS based on HLA I and HLA II binding predictions, transcript expression, variant allele frequency, and other criteria. The selection was manually reviewed per patient by a review board.

### Good manufacturing practice manufacturing of RNA–LPX

Manufacturing runs were conducted for all patients during 2019. No pre-specified target was set for turnaround time, as the individualized on-demand manufacturing process for neoantigen RNA–LPX was initiated as part of this clinical trial and continuously optimized during patient enrolment.

In short, two synthetic DNA fragments each coding up to ten neoantigen vaccine targets (SNVs and short insertions and deletions) connected by non-immunogenic glycine/serine linkers (30 bp long) were cloned into a starting vector^8^, containing the SEC^10^ (SEC, MRVMAPRTLILLLSGALALTETWAGS) and the MITD domain sequences^10^ (MITD, IVGIVAGLAVLAVVVIGAVVATVMCRRKSSGGKGGSYSQAASSDSAQGSDVSLTA) for fusion to mutated sequence concatemers for optimized routing to HLA class I and II pathways. The starting vectors also contained the FI-elements^9^ (FI, LVLHARNASCPFPVLGTPSLPRPRVPGMLPPPPAPLTTSASSRHL) and backbone sequence elements for improved RNA stability and translational efficiency. The DNA was linearized via PCR, spectrophotometrically quantified, and identified with Sanger sequencing, and subjected to in vitro transcription with T7 RNA polymerase as previously described in the presence of ATP, CTP, UTP, GTP and β-S-ARCA(D1) cap analogue in a cleanroom environment. RNA was purified using magnetic particles and integrity was assessed by gel electrophoresis and microfluidic capillary electrophoresis. Further analyses included determination of concentration, pH, osmolality, potency, and endotoxin level.

Liposomes with net cationic charges were used to complex the RNAs to form RNA–LPX. The cationic liposomes were manufactured using an adopted proprietary protocol^38^ based on the ethanol injection techniques^39^ from the cationic synthetic lipid (R)-*N*,*N*,*N* trimethyl-2-3-dioleyloxy-1-propanaminium chloride (R-DOTMA) and the phospholipid 1,2-dioleoyl-*sn*-glycero-3-phosphoethanolamine (DOPE) (Merck and Cie). Release analysis for the liposomes included determination of appearance, lipid concentration, RNase presence, particle size and polydispersity index, osmolality, pH, subvisible particles, pyrogen testing, and sterility. The RNA–LPX drug products were prepared in a good manufacturing practice manufacturing facility by first incubation of the individual RNAs with 14.6% (w:v) aqueous NaCl (Hospira) to arrive at the intended NaCl concentration in the RNA solution prior to mixing with the liposomes. Subsequent automated mixing of the pre-conditioned RNA with the liposomes was performed using a proprietary process, which allowed for accurate control of the mixing ratio between the two moieties. RNA–LPX were further diluted to the final drug product concentration by the addition of cryoprotectant containing customized buffers. The bulk drug product was filled into type 1 glass vials (Ompi). After inspection for visible particles, the drug product was frozen and delivered to the hospital as a concentrated dispersion for dilution. Release analysis for the RNA–LPX included determination of appearance, particle size and polydispersity, subvisible particles, RNA integrity, RNA content, pH, osmolality, endotoxin testing and sterility. For administration to the patient, the thawed drug product was diluted with 0.9% aqueous NaCl.

### Blood sampling for immunogenicity assays

For assessment of vaccine-induced immune responses, blood was sampled at baseline, before the fourth, sixth and eighth vaccine dose and 7–14 days after the eighth dose. After end of treatment, blood samples were taken 49–63 days after last dose. For patients actively participating in long-term follow-up, further samples were collected every 3 months after end of the main study phase in the first year and every 6 months in the second and third year. Patients not actively participating in long-term follow-up could donate blood for research purposes via a research project during routine visits at their clinic. PBMCs were isolated by Ficoll-Hypaque (Amersham Biosciences) density gradient centrifugation from peripheral blood or leukapheresis samples.

### Overlapping peptides

Synthetic 15mer peptides with 11 amino acid overlaps covering the neoantigen sequences, referred to as OLP pools (>70% purity), or 8–11mer peptides (>90% purity) were used in immunogenicity assessments. All synthetic peptides were purchased from JPT Peptide Technologies GmbH and dissolved in 10% DMSO to a final concentration of 3 mM.

### IVS of PBMCs

CD4^+^ and CD8^+^ T cells were isolated from cryopreserved PBMCs using microbeads (Miltenyi Biotec). Individual IVS cultures were set up using 15mer OLP pools encoding patient-specific neoantigen targets with up to five targets per expansion. For this, purified CD4^+^ T cells were expanded in the presence of fast dendritic cells, effector to target ratio (*E*:*T*) of 10:1, and the respective peptides. For the expansion of CD8^+^ T cells, purified CD8^+^ T cells were co-cultured with CD4-depleted PBMCs (E:T, 1:10 or 1:20) in the presence of IL-4 and GM-CSF (each 1.000 U ml^−1^) and the respective peptides. One day after starting the IVS, fresh culture medium containing 10 U ml^−1^ IL-2 (Proleukin S, Novartis), 5 ng ml^−1^ IL-15 (Peprotech) were added. CD8 IVS cultures additionally received IL-4 and GM-CSF (each 1,000 U ml^−1^). Seven days after setting up the IVS cultures, IL-2 was replenished (10 U ml^−1^). After 11 days of stimulation, cells were analysed via flow cytometry and used in ELISpot assay. Due to limited sample availability, IVS of PBMCs prior to ELISpot could only be performed for eight patients.

### IFNγ ELISpot

Multiscreen filter plates (Merck Millipore), precoated with IFNγ-specific antibodies (Mabtech), were washed with phosphate-buffered saline (PBS) and blocked with X-VIVO 15 (Lonza) containing 2% human serum albumin (CSL-Behring) for 1–5 h. Next, 0.5 × 10^5^ to 3.3 × 10^5^ effector cells per well were stimulated for 16–20 h either with peptides (ex vivo setting) or with autologous dendritic cells loaded with peptides (IVS samples). For analysis of ex vivo T cell responses, cryopreserved PBMCs were subjected to ELISpot after a resting period of 2–5 h at 37 °C. Alternatively, CD4- or CD8-depleted PBMCs were used as CD8 or CD4 effectors. All tests were performed in duplicate or triplicate and included positive controls (anti-CD3 (Mabtech; 1:1,000)), *Staphylococcus* enterotoxin B (Sigma-Aldrich) or PHA-L (Thermo Fisher). Bound IFNγ was visualized either using a secondary antibody directly conjugated with alkaline phosphatase (ELISpotPro kit, Mabtech; for ex vivo) or biotin-conjugated anti-IFNγ (Mabtech, 1:1,000) followed by incubation with ExtrAvidin–Alkaline Phosphatase (Sigma-Aldrich) for IVS samples. Next, plates were incubated with BCIP/NBT (5-bromo-4-chloro-3′-indolyl phosphate and nitro blue tetrazolium) substrate (ELISpotPro kit, Mabtech (ex vivo) or Sigma-Aldrich (IVS samples)). Plates were scanned using either an AID Classic Robot ELISpot Reader or CTL ImmunoSpot Series S6CORE analyser (ImmunoCapture Image Acquisition Software v.6.6) and analysed by AID ELISpot 7.0 software (AID Autoimmun Diagnostika) or ImmunoSpot Professional Software v.5.4. Spot counts were displayed as mean values of each duplicate or triplicate. In the ex vivo setting, peptide-stimulated spot counts for each sample were compared to effectors from the same sample incubated with medium only, as negative control using an in-house ELISpot data analysis tool (EDA), based on two statistical tests (distribution-free resampling) according to previous publications^40,41^. In case of a *P* value of <0.05 and minimum 7 counts, response to that peptide (neoantigen) was defined as a positive response. In the next step, ELISpot data analysis tool response calls for the pre- and post-vaccination samples were compared. Neoantigens with a positive response call only in the post-vaccination sample were classified as inducing a de novo response. Two neoantigens induced a significant increase in spot counts in the pre-vaccination sample. These spot counts were >11 fold higher in the post-vaccination sample (Extended Data Fig. 3, amplified responses highlighted in green boxes). These responses were classified as ‘amplified’. In the post-IVS setting, peptide-stimulated spot counts were compared to control peptide-loaded target cells using an in-house statistical analysis tool for pre- and post-vaccination samples. T cell vaccine responses were defined with an at least twofold increase in spot count after vaccination.

### Peptide–MHC multimer staining

**Extended Data Fig. 7:**
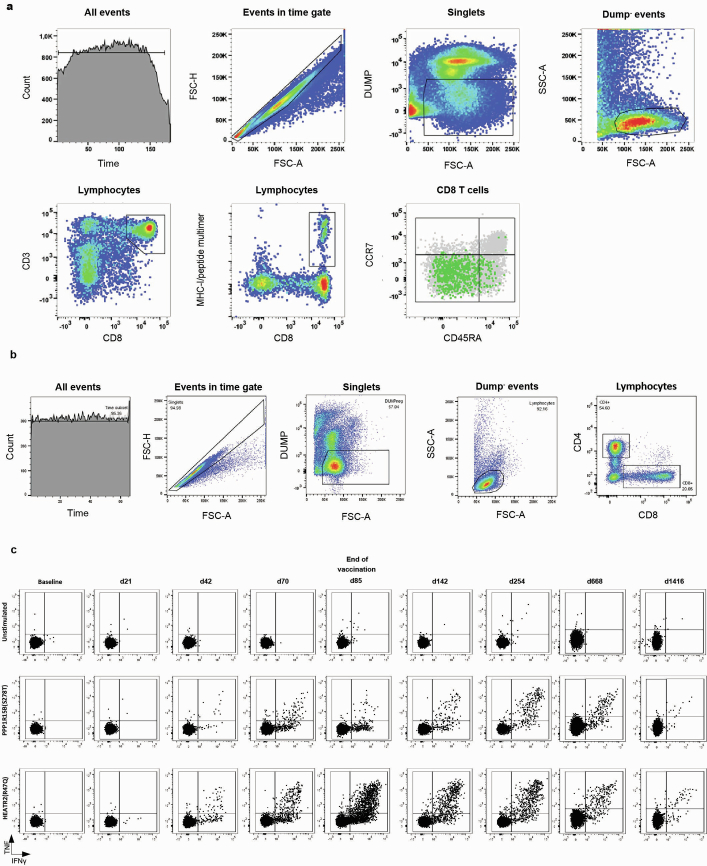
Flow cytometry analysis of PBMCs from P01. Gating strategy for analysis of neoantigen-specific T cells via multimer staining (**a**), as shown in Fig. 2f, g and h (**a**) or Intracellular Cytokine Staining (**b**), as shown in Fig. 2f right panel. Starting with events acquired with a constant flow stream and fluorescence intensity (events in time gate), single events were identified by plotting FSC-A vs. FSC-H (Singlets). In the next steps, viable and DUMP-negative events were identified (Dump^−^ events), within which population the lymphocytes were gated. Within lymphocytes, CD8 + T cells were gated for further analysis. Additionally (a), within the lymphocytes, multimer-positive CD8-positive events were gated (lower row, first two panels). Subsets of CD8 + T cells were gated based on total CD8 + T cells (shown in gray) by plotting CD45RA vs. CD197 (lower row, right panel). The resulting four subsets are central memory (CD45RA– CD197+), naïve (CD45RA+ CD197+), effector memory (CD45RA– CD197–) and TEMRA (CD45RA+ CD197–). The subsets in multimer+ events (highlighted in green) were gated analogously to the subsets gated for CD8 + T cells. (**c**) IFNγ and TNF production of CD8 + T cells in P01 after overnight incubation of PBMCs with medium alone (upper row) or corresponding peptides encoding for PPP1R15B(S278T) (middle row) and HEATR2(R47Q) (lower row) in the presence of GOLGI-inhibitors before staining.

**Extended Data Fig. 8:**
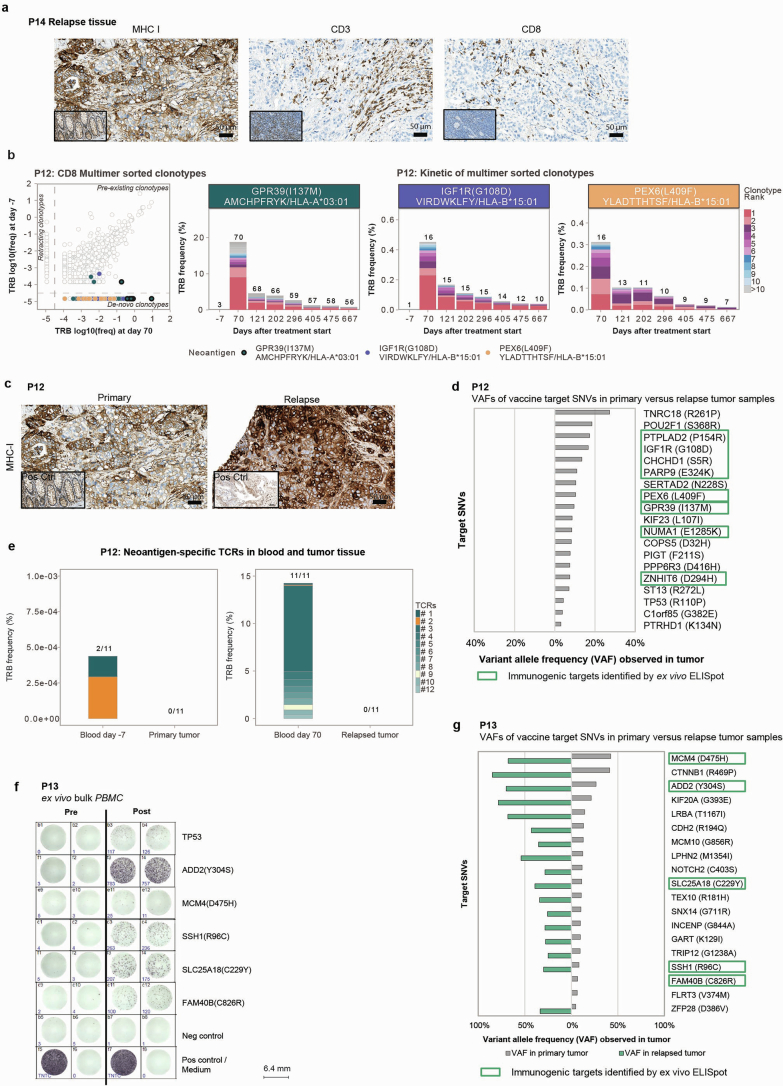
Characterization of T cells and tumor tissue of patients with disease recurrence. (**a**) Staining of post-vaccination FFPE section of relapse tumor tissue from P14, taken prior to initiating anti-PD-1 treatment. Inlets:staining of colon tissue as positive control. (**b**) Vaccine-induced expansion of neoantigen-specific T-cell clones identified via TCRseq of multimer-positive sorted cells. Comparison of pre- and post-vaccination blood samples (scatter plot). Neoantigen-specific clones for each of the three specificities are color-coded; clonotypes also identified via RevImMo analysis are circled in bold. Tracking of neoantigen-specific clones in bulk CD8 + TRB profiling data from longitudinal blood samples (bar graphs) with the top 10 clonotypes for each specificity in color. Number of clonotypes tracked in each sample are shown on top of each bar. **(c**) Staining of post-vaccination FFPE section of primary tumor used for vaccine design and relapse tumor tissue from P12. Inlets show staining of colon tissue as positive control. (**d**) Variant allele frequency of vaccine target SNVs in the pre- and post-vaccination tumor tissue of P12. VAFs were calculated according to read alignment depth of pooled sequencing replicates, with a minimum depth of three variant allele reads at the SNV position. (**e**) Cumulative frequency of neoantigen-specific TCRs in CD8+ cells in blood and tumor biopsy samples of P12. Shared clonotypes among pre-vaccination blood and primary tumor (left) and post-vaccination blood and relapsed tumor (right). The TRB CDR3 sequences were used for tracking in bulk profiling data. Numbers above bars: total number of neoantigen-specific TCRs. (**f**) Ex vivo T-cell responses for P13 by IFNγ ELISpot of total PBMCs. Negative control: PBMCs with medium, positive control: anti-CD3 (**g**) Variant allele frequency of vaccine target SNVs in the pre-vaccination and relapse tumor tissue of P13.

Mutation-specific CD8^+^ T cells were identified using fluorophore-coupled peptide/MHC (pMHC) multimers (ImmunAware) carrying 9-to-11-amino-acid peptides from immunogenic neoantigens. Potential minimal class I epitopes were identified by the NetMHCpan predictor (DTU Health Tech). Alternatively, peptide–MHC complexes were generated using easYmer technology (easYmer kit, ImmuneAware Aps), and complex formation was validated in a bead-based flow cytometry assay according to the manufacturer’s instructions. For tetramerization, streptavidin (SA)–fluorochrome conjugates were added: SA–BV421, SA–BV711, SA–PE, SA–PE-Cy7 or SA–APC (all BD Biosciences). Cells were stained for multimers first and then for cell surface markers, as follows (antibody clones and dilution in parentheses): CD28 (CD28.8, 1:25), CD197 (150503, 1:50), CD45RA (HI100, 1:100), CD3 (SK7, 1:100), CD16 (3G8, 1:100), CD14 (MφP9, 1:50), CD27 (L128, 1:50), CD279 (EH12, 1:25), CD127 (HIL-7R-M21, 1:50) and CD8 (RPA-T8, 1:50), all purchased from BD Biosciences; CD19 (HIB19) and CD4 (OKT4), from Biolegend. TCF-1 (C63D9, Cell Signaling) was stained intracellularly after permeabilization. We also carried out live-dead staining using 4′,6-diamidino-2-phenylindole (DAPI; BD) or fixable viability dyes eFluor 780 or eFluor 506 (eBioscience). Singlet, live, multimer-positive events were identified within CD3^+^ (or CD8^+^), CD4^−^CD14^−^CD16^−^CD19^−^ or CD3^+^ (or CD8^+^) CD4^−^ events (Extended Data Fig. 7a). For detection of antigen-specific T cells after IVS, single, live, CD3^+^, CD8^+^multimer^+^ lymphocytes were gated.

### Intracellular cytokine staining

PBMCs were incubated with peptides for around 16 h at 37 °C in the presence of brefeldin A and monensin. Cells were stained for viability (using fixable viability dye eFluor 780, eBioscience, 1:1667) and for surface markers CD8 (RPA-T8, 1:33), CD16 (3G8, 1:100), CD14 (MφP9, 1:50) (all from BD Biosciences), CD19 (HIB19, 1:50), or CD4 (OKT4, 1:25) (from Biolegend). After permeabilization, intracellular cytokine staining was performed using antibodies against IFNγ (B27, BD Biosciences, 1:100) and TNF (Mab11, BD, 1:167). IFNγ^+^ and TNF^+^ events were identified within the CD8^+^ and CD4^+^ cells pre-gated on single, live, and CD14^−^CD16^−^CD19^−^ (not used in all experiments) populations (Extended Data Fig. 7b).

Acquisition was performed on a LSR Fortessa SORP or FACSCanto II cell analyser (BD Biosciences) and analysed via FlowJo software (Tree Star).

### Tumour bulk TCR profiling

DNA was extracted from FFPE tumour tissue as described in ‘Next generation sequencing’. Sequencing and bioinformatic analysis to retrieve tumour TCR clonotypes was performed by iRepertoire using their proprietary pipelines.

### Blood bulk TCR sequencing and bioinformatic analysis

Total RNA was extracted from 3–5 × 10^5^ CD8^+^ or CD4^+^ T cells sorted by magnetic-activated cell sorting (MACS) using RNeasy Mini Spin Columns Qiagen kit. Total RNA was eluted in 30 μl water and the total yield was used for bulk TCR sequencing. Bulk TCR profiling libraries were generated using the SMARTer Human TCR a/b Profiling kit from Clontech Laboratories (now Takara Bio), according to the manufacturer’s manual. Sequencing of the library was performed on an Illumina MiSeq sequencer using the 600-cycle MiSeq Reagent Kit v.3 with paired-end, 2× 300 base pair reads. The data were demultiplexed using the Bcl2Fastq software (Illumina). Fasta sequences were edited performing a quality trimming step using the open-source trimmomatic v.0.36 software and then analysed with the open-source MiXCR software to annotate TCR sequences. TCR clonotypes with identical complementarity determining region 3 (CDR3) at the amino acid level were merged and their count and frequency values summed together. An in-house script was used to calculate repertoire statistics. Tracking of neoantigen-specific T cell clones in profiling data over time and in tumour tissue was performed using TRB CDR3 chain amino acid sequence as a molecular identifier.

### Single-cell sequencing and read processing

MACS-sorted CD8^+^ or CD4^+^ cells were washed once with 1 ml Dulbecco’s PBS (DPBS) with 0.04% BSA using a wide-bore tip centrifuged at 300*g* for 4 min, and resuspended at 5.3 × 10^5^ cells per ml. Twenty thousand cells (37.8 µl) were then transferred into a fresh tube and libraries for TCR VDJ and gene expression analysis prepared following the manufacturer’s instruction (User Guide Chromium Next GEM Single-Cell V(J)J Reagent Kits v.1.1, 10x Genomics, CG000207 Rev. D). Sequencing of the TCR libraries were performed on an Illumina MiSeq sequencer using the MiSeq Reagent Kit, 600 cycles, v.3 with paired-end, 2× 150 base pair reads. Sequencing of the 5′ gene expression library was performed on an Illumina NovaSeq sequencer using the NextSeq 1000/2000 P2 reagents, 100 cycles, v.3 with paired-end, 26× 91 base pair reads. Raw sequencing data were processed using the Cell Ranger software (10x Genomics) to generate clonotype data and raw count matrices of gene expression. For the VDJ data cell ranger, output files were then reshaped to a compatible format with our in-house data analysis pipeline and filtered to keep only clonotypes that contain both a TRA and a TRB chain. Clonotypes with two TRB chains were discarded.

### RevImMo analysis

Bulk TCR profiling data generated from pre- and post-vaccination CD8^+^ (and CD4^+^) T cells were used to calculate clonal enrichment post-treatment for each TRB CDR3 clonotype. For de novo clones, which were not detected pre-treatment, treatment-induced enrichment was calculated by assigning an arbitrary frequency value corresponding to the lowest frequency detected in the corresponding repertoire. Clones present at both time points were termed pre-existing. Single-cell TCR profiling data from post-treatment CD8^+^ (or CD4^+^) T cells were used to retrieve the paired TRA/TRB information for each TRB clonotype. Only TRB clonotypes with paired information were ranked by enrichment and used for clone selection (Fig. 3a). Ten to fifteen each of the most enriched pre-existing and de novo TCRs were selected for further validation. Synthesized TCR V(D)J genes of the selected candidates were cloned into the pST1-TRAC/TRBC1/TRBC2 vector backbones by Twist Bioscience. DNA template manufacturing PCR was performed to amplify linearized templates of the cloned TRA and TRB chains which were then used to produce in vitro transcribed RNAs for validation of their specificity. The vaccine neoantigen specificity and functionality of the selected TCR candidates was evaluated using an optimized Jurkat-NFAT-reporter assay. The genetically engineered Jurkat T cell line expresses luciferase as a reporter, driven by an NFAT-response element (NFAT-RE), which is induced by the TCR-specific signalling cascade. NFAT–TCR/CD3 effector cells were purchased from Promega as cryopreserved cells. Reauthentication of cell lines was performed by short tandem repeat (STR) profiling at ATCC and Eurofins. All used cell lines tested negative for mycoplasma contamination. Optimization of used reporter cells was conducted by CRISPR–Cas9-mediated knockout of the endogenously expressed TCR and by transposon-based stable insertion of the CD8 co-receptor (alpha and beta chain). After electroporation of TRA- and TRB-encoding IVT-RNAs and a 20 h incubation period, 2 × 10^4^ Jurkat cells were co-cultured with K562 cells or autologous CD14^+^ monocyte cells at a 5:1 ratio, in a 384-well plate with 25 μl medium (RPMI1640 + 10% non-heat inactivated FBS) per well. Prior to co-culture, the K562 cells were transfected with IVT-RNA encoding the patient’s HLA alleles and loaded with neoantigen target peptide pools. The autologous CD14 cells were loaded with neoantigen target peptide pools. For all assays, patient-specific neoantigen targets were tested on all patient-specific HLA alleles (Extended Data Fig. 4). After 6 h, an equal volume (25 μl) of luciferin (Bio-Glo, Promega) was added to each well and the luciferase activity was measured using a luminescence plate reader. The measured luminescence signal in the different wells corresponded to the level of TCR-mediated activation in the Jurkat cells. For each TCR, fold change of luminescence compared with the TCR-transfected effectors-only control was calculated and a cut-off of twofold change was used to determine specific TCRs. In selected cases, negatively tested TCRs were subsequently evaluated by multimer-based staining experiments. CD8 TCR no. 18 from P1 was determined positive using this method.

### Single-cell gene expression analysis

Count matrices were analysed using Seurat software v.5.1.0^42^. Cells were associated with the corresponding VDJ data using cell barcodes and only cells with an associated VDJ clonotype were kept. To eliminate low quality cells and doublets, cells expressing two different TRB chains, more than 6,000 and less than 100 unique genes and with a percentage of mitochondrial unique molecular identifiers, more than 10% were excluded from the analysis. Filtered data were then normalized and scaled using the SCTransform function of the Seurat package^43^ with default parameters and regressing the percentage of mitochondrial counts. Data filtering and normalization was performed for each sample separately. Normalized data from different time points and patients were then integrated using Harmony 1.2.1 using RunHarmony function. After performing principal component analysis, the first 14 principal components were selected by examining the ‘elbow plot’. Clustering analysis was performed using the FindNeighbors and FindClusters functions with a resolution of 0.5. Cluster identity was assigned based on differential expressed genes identified using FindAllMarkers Seurat function. Based on TRB CDR3 sequences, neoantigen-specific cells were identified and selected for further analysis. These cells were reanalysed as described above using the first 10 principal components and a resolution of 0.3. After differential gene expression analysis, clusters with similar phenotype were merged.

### Single-cell TCR sequencing of multimer-positive cells

Three peptide–HLA dextramer reagents were prepared by mixing each 2.16 pmol peptide-loaded HLA monomer (Immudex) with 0.48 µl ULoad dCODE dextramer, conjugated to a specific DNA barcode (Immudex). Peptide–HLA monomers and dextramers were incubated for 30 min in the dark at 4 °C. An empty dextramer was used as a control. For multimer staining, 0.8 µl 100 µM biotin was combined with assembled peptide–HLA and control dextramers for pooled staining of PBMCs. PBMCs were resuspended and washed in MACS buffer. A total of 10 × 10^6^ viable PBMCs was resuspended in PBS/2% human serum albumin (HSA) and stained with 16.6 µl dextramer pool after adding 5 µl Human TruStain FcX Block (Biolegend) in a total volume of 50 µl. Cells were stained for 10 min at 4 °C in the dark. Fifty microlitres antibody surface staining mix was added (BV480 mouse anti-human CD8, BB515 mouse anti-human CD4, APC-eFlour780 mouse anti-human CD16/CD14/CD19, fixable viability dye eFlour780). Cells were stained with antibody mix for 30 min at 4 °C. Cells were then washed 3 times with each 1 ml PBS/2% HSA and resuspended in a volume of 500 µl PBS/2% HSA. Multimer-positive cells were sorted by fluorescence-activated cell sorting (FACS) by first gating the dump channel (eFlour780)-negative, CD8-positive lymphocyte population. Dextramer backbones were phycoerythrin (PE)-labelled such that multimer-specific CD8 T cells were isolated as the PE-positive population. Sorted multimer-positive T cells were centrifuged and resuspended in a volume of 50 µl in PBS/0.04% BSA. Cell concentration was determined, and cells were diluted to a final concentration of 5.3 × 10^5^ cells per ml. Single-cell RNA-seq was performed as described above. VDJ and Feature Barcoding libraries were produced as described by the manufacturer’s instructions (Chromium Next GEM Single-Cell 5′ Reagent Kits v.2 [Dual Index], 10x Genomics, CG000511 Rev. B). Data Processing of VDJ libraries was performed as described above. Feature barcoding libraries were processed using Cell Ranger software to assign multimer barcodes to sequenced cells. Antigen specificity of individual TCR clones was determined using in-house scripts after merging VDJ and feature barcoding data based on cell barcodes.

### Immunohistochemical staining

To determine the expression of CD3, CD8 and MHC-I, 3- to 4-µm-thick sections of FFPE tumour tissue were analysed.

Staining was performed on the Ventana Discovery Ultra platform. For antigen retrieval Ultra CC1 was used for 64 min. Slides were afterwards incubated for 60 min at 37 °C with anti-human CD3 (2GV6; Roche Diagnostics at a ready-to-use dilution), anti-human CD8 (SP57; Roche Diagnostics, 1:100) and MHC-I (EPR1394Y; Abcam, 1:500) followed by secondary antibody OmniMap anti-rabbit HRP (Roche Diagnostics, at a ready-to-use dilution) for 32 min at 37 °C. For visualization, ChomoMap DAB Kit (Roche Diagnostics) was used followed by counterstain (Hematoxylin II, Bluing reagent; Roche Diagnostics). Slides were scanned (Axio.Scan; Zeiss) and manually analysed.

### Differential gene expression analysis, GSEA and variant allele frequency calculation

Whole-exome sequencing and RNA-seq data for P12 and P13 from post-treatment samples were analysed using the bioinformatics software pipeline. Differential expression analysis on RNA-seq data was performed with R (v.4.0.2) using tximport (v.1.18) to load read counts generated by sailfish and summarize transcript read counts by genes. Summarized counts were imported into DESeq2 (v.1.30)^44^ for differential expression testing according to instructions on the package vignette, only considering genes that were covered by at least ten reads across all samples. Differential expression testing results were exported with a significance cut-off of 0.01 (after false discovery correction using the Benjamini and Hochberg method^45^) and shrunken log fold change values were calculated. GSEA was performed using fgsea 1.20.0^46^ R package on MSigDB v.7.5.1^47^ KEGG canonical pathways. Pre- versus post-treatment tumour differential expressed genes were ranked based on the Wald statistic value and used as input of the fgsa function performing 1,000 permutations. Variant allele frequencies for somatic SNVs called in the pre-treatment tumour were determined from the aligned tumour BAM files using pysam (v.0.15.4)^48–50^.

### Kaplan–Meier time-to-event analysis for disease-free survival probability

Disease-free survival was a non-protocol-specified analysis performed for this report. Disease-free survival was defined as the time from first vaccine dose to cancer recurrence or death from any cause. Imaging after initial (neo)adjuvant treatment was only performed upon suspected progression, which may overestimate disease-free survival. Date of last visit/contact served as the date of censoring. The analysis was performed using SAS v.9.4.

### Materials availability

Materials are available from the authors under a material transfer agreement with BioNTech. This work is licensed under a Creative Commons Attribution 4.0 International (CC BY 4.0) license, which permits unrestricted use, distribution, and reproduction in any medium, provided the original work is properly cited. To view a copy of this license, visit http://creativecommons.org/licenses/by/4.0/. This license does not apply to figures/photos/artwork or other content included in the article that is credited to a third party; obtain authorization from the rights holder before using such material.

### Reporting summary

Further information on research design is available in the Nature Portfolio Reporting Summary linked to this article.

## Online content

Any methods, additional references, Nature Portfolio reporting summaries, source data, extended data, supplementary information, acknowledgements, peer review information; details of author contributions and competing interests; and statements of data and code availability are available at 10.1038/s41586-025-10004-2.

## Supplementary information

Supplementary Information A single merged pdf containing a title page, contents page and Supplementary Tables 1 and 2. Supplementary Table 1. Immunogenicity of indels used as neoantigen vaccine targets; Supplementary Table 2. Neoantigen-specific TCR-α/β chains cloned from single T cells of three patients.

Reporting Summary

Supplementary Data 1 An Excel file providing an overview of T cell responses at the target level.

Supplementary Data 2 An Excel file providing details of differentially expressed genes in post- versus pre-treatment tumour tissue from P13.

Supplementary Data 3 An Excel file showing gene set enrichment analysis of MSigDB KEGG pathways of post- versus pre-treatment tumour tissue from P13.

## Figures and Tables

**Extended Data Table 1:**
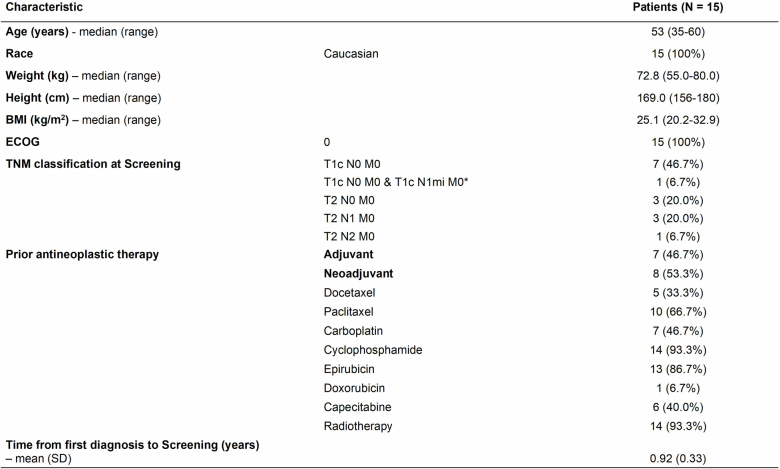
Demographic and clinical characteristics

Fifteen patients consented for the neoantigen vaccine part of this trial. One patient discontinued after 3 vaccine doses due to treatment-emergent adverse events (hypotension, grade 3; nausea, grade 2; chills, grade 1) and was lost to immunogenicity sample collection and follow-up; 14 patients were evaluable. *Patient with bilateral breast cancer; both TNM classifications are presented.

BMI, body mass index; ECOG PS, Eastern Clinical Oncology Group performance status.

**Extended Data Table 2:**
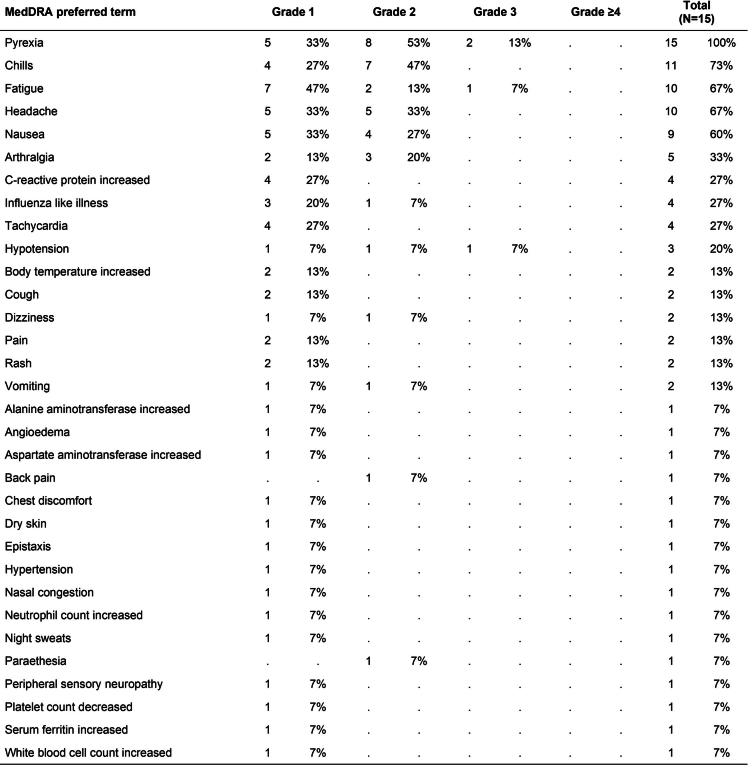
Related treatment-emergent adverse events

Patients with at least one TEAE considered related to treatment, worst severity is presented as n, (%).

## Data Availability

All data associated with this study are present in the paper or supplementary materials.
