# Supplementary material for: Individualized mRNA vaccines evoke durable T cell immunity in adjuvant TNBC
Source: Nature. 2026 Feb 18;651(8107):1088–96. doi: 10.1038/s41586-025-10004-2 (PMC13017525; doi:10.1038/s41586-025-10004-2)
Supplement: Supplementary file 1 — A single merged pdf containing a title page, contents page and Supplementary Tables 1 and 2. Supplementary Table 1. Immunogenicity of indels used as neoantigen vaccine targets; Supplementary Table 2. Neoantigen-specific TCR-α/β chains cloned from single T cells of three patients. [file 41586_2025_10004_MOESM1_ESM.pdf]

nature portfolio

https://doi.org/10.1038/s41586-025-10004-2

Supplementary information

Individualized mRNA vaccines evoke durable T cell immunity in adjuvant TNBC

In the format provided by the authors and unedited

Supplementary Information

Individualized mRNA vaccine-induced neoantigen-specific T-cell responses in post (neo)adjuvant TNBC

Authors: U. Sahin[1,2,7†*], M. Schmidt[3†], E. Derhovanessian[1], A. Cortini[1], I. Vogler[1], T. Omokoko[1], E. Godehardt[1], S. Attig[2], S. Newrzela[1], J. Grützner[1], N. Bidmon[1], S. Bolte[1], S. Brachtendorf[1], T. Stuhlmann[1], D. Langer[1], D. Brüne[1], J. Blake[1], A. Feldner[1], H. Lindman[4], A. Schneeweiss[5], M. Eichbaum[6], Ö. Türeci[1,7]

Affiliations:

[1]BioNTech Group; Mainz, Germany.

[2]TRON gGmbH; Mainz, Germany.

[3]Department of Obstetrics and Gynecology, University Medical Center of the Johannes Gutenberg-University; Mainz, Germany.

[4]Department of Immunology, Genetics and Pathology, Uppsala University Hospital; Uppsala, Sweden.

[5]Division Gynecologic Oncology, National Center for Tumor Diseases, University Hospital and German Cancer Research Center; Heidelberg, Germany.

[6]Klinik für Frauenheilkunde und Geburtshilfe, Helios Dr. Horst Schmidt Kliniken Wiesbaden; Wiesbaden, Germany.

[7]HI-TRON Mainz, Mainz, Germany.

† co-first author

*Corresponding author. Email: ugur.sahin@biontech.de

Supplementary Information Contents

	Page
**Supplementary Table 1. Immunogenicity of INDELs used as neoantigen vaccine targets**	3
**Supplementary Table 2. Neoantigen-specific TCR-α/β chains cloned from single T cells of three patients**	4–5

Supplementary tables

Supplementary Table 1. Immunogenicity of INDELs used as neoantigen vaccine targets

Patient	Gene	INDEL sequence	Effect	Post-IVS CD4+ response	Post-IVS CD+8 response	*Ex vivo* response
P01	TP53	SGNLLGRNSFEVRVCAACPGRDRRTEEENLR	In-frame insertion	*de novo*	-	-
P03	KDM5A	L**VPDDERSVQHAEPHVFSLLSHVPVILSGLYVSTIQLICAPAPCRRNVL**	Frameshift deletion	-	*de novo**	-
	VAMP1	CAIIVVVIVSKYR**GSCWWGEEVGRSWS**	Loss of stop codon by substitution; readthrough mutation	*de novo*	-	-
P05	DUSP1	**CSTPLYDQGGNPALSVPGQCVSRFPQGHAGCLGHHCLDQRLSQLSQPF**	Frameshift deletion	-	-	-
	POLI	**LFSRNHTTDSHKQQTLMKDLQKIESQILLMRKLLSLLTLILKFSMNYQKQ**	Frameshift deletion	n.e.	amplified	*de novo*
	MALT1	DLEHQGTYWCHVYNDRDSKKVEIIIGRTDEA	In-frame deletion	n.e.	-	-
P07	FAM116A	EDLQGILLKTGMT**LYLPRFSSQKGLCI**	Substitution; readthrough mutation	n.d.	n.d.	*de novo*
P08	ZNF331	**NVGRPSIVAITSLSTRESTQAKPRINVRSVGRLSFMDRAS**	Frameshift insertion	-	-	-
	RAB3GAP2	LKTVKPLSLFDSKGK**KCIFQRPNFNSVIT**	Frameshift deletion	-	-	-
P09	WDFY3	KSSSSYHCSIIAMKT**LT**RHDYIFKDVFREVG	In-frame deletion	n.d.	n.d.	-
	FLRT2	VSLNNDQLLKGDFRLQP**FTPQMGALITQTAISPTTCDTATAACQ**	Frameshift deletion	n.d.	n.d.	-
P11	EFCAB4A	RRRESEHEREVRALY**EEQ**LREQSRRPPSQVG	In-frame deletion	n.d.	n.d.	-
P12	PLXNB2	NIDSKLHVTLYNCSF**AAATAACAGPLTPTTGVRGAGARAGACMRP**	Frameshift deletion	n.d.	n.d.	-
P13	TP53	APAPAPSWPLS**FFCPFPENLPGQLRFPSGLLAFWDSQVCDLHVLPCPQQD**	Frameshift insertion	n.d.	n.d.	*de novo*
P14	CTNNA1	**PDPGSQELDECCGADSEGILRRLYQIPKVTGYGFPQPSCCVMEDEGTREK**	Frameshift deletion	-	-	-
	CTNNA1	**PAGLPATHRPLLPPAEHLQQGQGRGAESRRGACCLWEL**	Frameshift deletion	-	-	-

Vaccine-encoded INDELs and T-cell responses identified by post-IVS and *ex vivo* ELISpot analysis. * two different HLA alleles were found to be restriction elements of CD8+ T cells against this INDEL (Fig. S2D). New polypeptide sequences are highlighted in bold, amino acids affected by an in-frame frameshift are underscored. - = no response, n.d. = not done, n.e. = not evaluable, INDEL = insertion/deletion.

Supplementary Table 2. Neoantigen-specific TCR-α/β chains cloned from single T cells of three patients

Patient	Mutation	TCR name	TRA	TRB	HLA restriction	Recognized peptide	Pre-existing
P01	HEATR2(R47Q)	TCRCD8-P01-#1	V6 J32*03 C	V14 D J1-6*02 C1	B*07:02	KPGRQRALEAL	+
	HEATR2(R47Q)	TCRCD8-P01-#3	V6 J21 C	V5-1 D J2-1 C2	B*07:02	KPGRQRALEAL	+
	HEATR2(R47Q)	TCRCD8-P01-#14	V19 J41 C	V7-9*03 D J2-2 C2	B*07:02	KPGRQRALEAL	+
	HEATR2(R47Q)	TCRCD8-P01-#17	V21 J24 C	V15*02 D J2-3 C2	B*07:02	KPGRQRALEAL	-
	HEATR2(R47Q)	TCRCD8-P01-#21	V6 J28 C	V28 D J1-2 C1	B*07:02	KPGRQRALEAL	-
	HEATR2(R47Q)	TCRCD8-P01-#25	V6 J45 C	V2 D J2-7 C2	B*07:02	KPGRQRALEAL	-
	PPP1R15B(S278T)	TCRCD8-P01-#11	V19 J28 C	V20-1 D J2-1 C2	B*58:01	LSAELIPATW	+
	PPP1R15B(S278T)	TCRCD8-P01-#13	V19 J36 C	V4-1 D J2-7 C2	B*58:01	LSAELIPATW	-
	PPP1R15B(S278T)	TCRCD8-P01-#16	V14/DV4 J46 C	V2 D J1-5 C1	B*58:01	LSAELIPATW	-
	PPP1R15B(S278T)	TCRCD8-P01-#18	V38-1 J29 C	V4-1 D J1-4 C1	B*58:01	LSAELIPATW	-
	PPP1R15B(S278T)	TCRCD8-P01-#19	V26-1 J49 C	V7-9*03 D J2-7 C2	B*58:01	LSAELIPATW	-
	PPP1R15B(S278T)	TCRCD8-P01-#20	V26-1 J49 C	V2 D J1-1 C1	B*58:01	LSAELIPATW	-
	PPP1R15B(S278T)	TCRCD8-P01-#22	V19 J7 C	V4-1 D J2-7 C2	B*58:01	LSAELIPATW	-
	PTCD3(I448T)	TCRCD8-P01-#23	V12-3 J34 C	V28 D J2-3 C2	B*58:01	KTGDNWKFTG	-
	PTCD3(I448T)	TCRCD8-P01-#24	V17 J45 C	V4-1 D J2-5 C2	B*58:01	KTGDNWKFTG	-
	PTCD3(I448T)	TCRCD8-P01-#26	V5 J16 C	V5-8 D J2-7 C2	B*58:01	KTGDNWKFTG	+
	EIF4G3(R795Q)	TCRCD4-P01-#14	V13-1 J6 C	V9 D J2-2 C2	DRB1*08:04	ENIKTQELFRKVQS	+
	PPP1R15B(S278T)	TCRCD4-P01-#19	V8-4 J54 C	V18 D J2-1 C2	n.d.	n.d.	-
P12	GPR39(I173M)	TCRCD8-P12-#1	V35 J41 C	V6-2 D J1-2 C1	A*03:01	-	+
	GPR39(I173M)	TCRCD8-P12-#3	V35 J28 C	V11-2 D J2-3 C2	A*03:01	-	-
	GPR39(I173M)	TCRCD8-P12-#4	V38-1 J39 C	V3-1 D J2-3 C2	A*03:01	-	-
	GPR39(I173M)	TCRCD8-P12-#5	V10 J20 C	V27 D J2-7 C2	A*03:01	-	-
	GPR39(I173M)	TCRCD8-P12-#6	V8-6*02 J54 C	V20-1 D J2-7 C2	A*03:01	-	-
	GPR39(I173M)	TCRCD8-P12-#7	V9-2*02 J12 C	V28 D J2-5 C2	A*03:01	-	-
	GPR39(I173M)	TCRCD8-P12-#8	V8-6*02 J20 C	V20-1 D J2-1 C2	A*03:01	-	-
	GPR39(I173M)	TCRCD8-P12-#10	V12-1 J9 C	V19 D J2-5 C2	A*03:01	-	-
	GPR39(I173M)	TCRCD8-P12-#12	V27 J9 C	V6-1 D J2-1 C2	A*03:01	-	-
	PEX6(L409F)	TCRCD8-P12-#2	V17 J56 C	V6-6*02 D J2-3 C2	C*03:03	-	+
	PEX6(L409F)	TCRCD8-P12-#9	V24 J49 C	V6-2 D J2-2 C2	C*03:03	-	-
P13	ADD2(Y304S)	TCRCD8-P13-#1	V35 J58 C	V13 D J2-7 C2	A*68:01	-	+
	ADD2(Y304S)	TCRCD8-P13-#10	V9-2*02 J7C	V12-4 D J1-1 C1	A*68:01	-	-
	ADD2(Y304S)	TCRCD8-P13-#11	V14/DV4 J31 C	V2 D J2-5 C2	A*68:01	-	-
	SLC25A18(C229Y)	TCRCD8-P13-#9	V5 J39 C	V12-4 D J2-2 C2	A*02:01	-	+
	SLC25A18(C229Y)	TCRCD8-P13-#17	V20 J40 C	V12-4 D J2-3 C2	A*02:01	-	-
	SLC25A18(C229Y)	TCRCD8-P13-#18	V12-1 J23 C	V28 D J2-1 C2	A*02:01	-	-

SSH1(R96C)	TCRCD8-P13-#2	V29/DV5 J48 C	V27 D J1-5 C1	B*14:02	-	+
SSH1(R96C)	TCRCD8-P13-#19	V26-2 J48 C	V27 D J2-1 C2	B*14:02	-	-
SSH1(R96C)	TCRCD8-P13-#20§	V19 J44 C	V19 D J1-4 C1	B*14:02	-	-
TP53	TCRCD8-P13-#12	V14/DV4 J7 C	V5-1 D J1-1 C1	B*14:02	-	-

The TCR V(D)J genes are indicated in IMGT-nomenclature. V, variable; D, diversity; J, joining; C, constant; n.d.; not determined; -, not investigated.

§ TCR_{CD8}-P13-#20 was not selected based on vaccine-induced clonotype enrichment in the periphery, but due to its presence in relapsing tumor tissue.
