# Supplementary material for: Individualized mRNA vaccines evoke durable T cell immunity in adjuvant TNBC
Source: Nature. 2026 Feb 18;651(8107):1088–96. doi: 10.1038/s41586-025-10004-2 (PMC13017525; doi:10.1038/s41586-025-10004-2)
Supplement: Supplementary file 2 — Reporting Summary [file 41586_2025_10004_MOESM2_ESM.pdf]

nature portfolio

Corresponding author(s): Ugur Sahin

Last updated by author(s): 07 11 2025

Reporting Summary

Nature Portfolio wishes to improve the reproducibility of the work that we publish. This form provides structure for consistency and transparency in reporting. For further information on Nature Portfolio policies, see our Editorial Policies and the Editorial Policy Checklist.

Statistics

For all statistical analyses, confirm that the following items are present in the figure legend, table legend, main text, or Methods section.

n/a	Confirmed	
☐	☒	The exact sample size (*n*) for each experimental group/condition, given as a discrete number and unit of measurement
☐	☒	A statement on whether measurements were taken from distinct samples or whether the same sample was measured repeatedly
☐	☒	The statistical test(s) used AND whether they are one- or two-sided<br>*Only common tests should be described solely by name; describe more complex techniques in the Methods section.*
☒	☐	A description of all covariates tested
☒	☐	A description of any assumptions or corrections, such as tests of normality and adjustment for multiple comparisons
☐	☒	A full description of the statistical parameters including central tendency (e.g. means) or other basic estimates (e.g. regression coefficient) AND variation (e.g. standard deviation) or associated estimates of uncertainty (e.g. confidence intervals)
☒	☐	For null hypothesis testing, the test statistic (e.g. *F*, *t*, *r*) with confidence intervals, effect sizes, degrees of freedom and *P* value noted<br>*Give P values as exact values whenever suitable.*
☒	☐	For Bayesian analysis, information on the choice of priors and Markov chain Monte Carlo settings
☒	☐	For hierarchical and complex designs, identification of the appropriate level for tests and full reporting of outcomes
☒	☐	Estimates of effect sizes (e.g. Cohen's *d*, Pearson's *r*), indicating how they were calculated

Our web collection on statistics for biologists contains articles on many of the points above.

Software and code

Policy information about availability of computer code

Data collection: agCapture 3.4.2.6

Data analysis

SAS v9.4. was used as programming software.
Genomics-related data analysis steps were coordinated by proprietary bioinformatic pipeline (BioNTech) implemented in the Python programming language; SAMtools was used to identify germline variants in the region of the mutated peptides. Sailfish (v.0.7.6) and STAR (v2.4.2a) were used to align RNA reads to the hg19 reference genome. NGSCheckMate (v.1.0) was used to confirm the identical patient as origin for all DNA and RNA libraries analyzed under the same patient ID.
HLA binding affinity was predicted via the IEDB T-cell prediction tools (v2.13), and an algorithm implemented in R (r-project.org) was used to select neoantigen vaccine targets.
For ELISPot, plates were scanned using either an AID Classic Robot ELISpot Reader or CTL's ImmunoSpot® Series S6CORE analyzer (ImmunoCaptureTM Image Acquisition Software V6.6) and analyzed by AID ELISpot 7.0 software (AID Autoimmun Diagnostika) or ImmunoSpot® Professional Software V5.4. An in-house ELISpot data analysis tool was subsequently used to analyse ELISpot data.
For single-cell sequencing, raw sequencing data were processed using the Cell Ranger software (10x Genomics) to generate clonotype data and raw count matrices of gene expression. For single-cell Gene Expression Analysis, count matrices were analyzed using Seurat software v5.1.0. Filtered data were normalized and scaled using the SCTransform function of the Seurat package, and normalized data from different time points and patients were integrated using Harmony 1.2.1.
Differential expression analysis on RNA-seq data was performed with R (v.4.0.2) using tximport (v.1.18) to load read counts generated by sailfish and summarize transcript read counts by genes. GSEA was performed using fgsea 1.20.0 47 R package on MSigDB v7.5.1, and variant allele frequencies for somatic SNVs called in the pre-treatment tumor were determined from the aligned tumor BAM files using pysam (v.0.15.4).
Flow cytometry data was analysed using FlowJo software version 10 (FlowJo LLC, BD Biosciences).

For manuscripts utilizing custom algorithms or software that are central to the research but not yet described in published literature, software must be made available to editors and reviewers. We strongly encourage code deposition in a community repository (e.g. GitHub). See the Nature Portfolio guidelines for submitting code & software for further information.

Data

Policy information about availability of data

All manuscripts must include a data availability statement. This statement should provide the following information, where applicable:

- Accession codes, unique identifiers, or web links for publicly available datasets
- A description of any restrictions on data availability
- For clinical datasets or third party data, please ensure that the statement adheres to our policy

All data associated with this study are present in the paper or supplementary materials, with the "minimum dataset" supplied as an excel file in addition to the paper.

Research involving human participants, their data, or biological material

Policy information about studies with human participants or human data. See also policy information about sex, gender (identity/presentation), and sexual orientation and race, ethnicity and racism.

Reporting on sex and gender

Sex is reported per protocol

Reporting on race, ethnicity, or other socially relevant groupings

Race is reported in Table S1. Analyses were not based on race as all participants were caucasian.

Population characteristics

Population characteristics are reported in table S1.

Recruitment

Patients were recruited at a total of four sites in Germany and Sweden, with patients screened and enrolled following completion of (neo)adjuvant chemotherapy. Recruitment to the two cohorts recruiting the neoantigen vaccine occured subsequently for each cohort, after at least six patients had been treated in a trial arm treating patients with TAA-vaccine only. Attempts were made to limit bias through study design to ensure a representative population of patients with early-stage TNBC.
Eligible adult female patients had histologically confirmed invasive adenocarcinoma TNBC, pT1cN0M0 – anyTanyNM0, treated with neoadjuvant or adjuvant chemotherapy, and an ECOG performance status (PS) of 0-1. Tumor biopsies were screened for expression of TAAs and neoantigens. Patients with recurrence of breast cancer prior to the start of study treatment were excluded.

Ethics oversight

This clinical trial was approved by the independent ethics committees (Ethik-Kommission of the Landesärztekammer Rheinland Pfalz, Mainz, Germany and Regionala Etikprövningsnämnden, Uppsala, Sweden) and the competent regulatory authorities (Paul-Ehrlich Institute, Langen, Germany and Medical Products Agency, Uppsala, Sweden).

Note that full information on the approval of the study protocol must also be provided in the manuscript.

Field-specific reporting

Please select the one below that is the best fit for your research. If you are not sure, read the appropriate sections before making your selection.

☒ Life sciences ☐ Behavioural & social sciences ☐ Ecological, evolutionary & environmental sciences

For a reference copy of the document with all sections, see nature.com/documents/nr-reporting-summary-flat.pdf

Life sciences study design

All studies must disclose on these points even when the disclosure is negative.

Sample size	This study recruited 42 patients, with 15 patients treated with neoantigen vaccine. 15 patients were planned as any (adverse) events with incidence of 20% or higher could be observed at least once with probability of at least 95%.
Data exclusions	One patient who discontinued treatment due to treatment-emergent adverse events after a total of three vaccinations was included in the safety analysis set only. 27 patients treated only with TAA vaccine in this clinical trial are not included in this report.
Replication	Replication is not applicable to this phase 1 clinical trial.
Randomization	As this first-in-human clinical trial did not involve formal comparison, patients were not assigned to trial arms or dose cohorts according to a randomization plan. When an eligible patient was identified at a trial site and after informed consent was given, the investigator sent a patient screening letter to sponsor. The sponsor then assigned the patient to a trial arm and dose cohort based on cohort recruitment status and tumor/DNA/RNA sequencing and manufacturing slot availability.
Blinding	This was an open-label study.

Reporting for specific materials, systems and methods

We require information from authors about some types of materials, experimental systems and methods used in many studies. Here, indicate whether each material, system or method listed is relevant to your study. If you are not sure if a list item applies to your research, read the appropriate section before selecting a response.

Materials & experimental systems

n/a	Involved in the study	
☐	☒	Antibodies
☐	☒	Eukaryotic cell lines
☒	☐	Palaeontology and archaeology
☒	☐	Animals and other organisms
☐	☒	Clinical data
☒	☐	Dual use research of concern
☒	☐	Plants

Methods

n/a	Involved in the study	
☒	☐	ChIP-seq
☐	☒	Flow cytometry
☒	☐	MRI-based neuroimaging

Antibodies

Antibodies used

Used antibodies (specificity/ fluorochrome/clone/manufacturer/catalogue number/dilution/lots):

Reagents for IFN-γ ELISpot
Primary anti-human IFN-γ antibody/clone Mab 1-D1K/MABTECH/3420-3-1000/1:1000/ 93.3, 95.2, 96.1, 96.2, 106.1, 107.2
Secondary anti-human IFN-γ antibody/clone 7-B6-1 (biotinylated)/MABTECH/3420-6-1000/ 1:1000/ 50.2, 53.1, 54.1, 54.2, 54.5
aCD3/clone CD3-2/1:1000

Mabtech ELISpot kit (human IFN-γ (ALP)/clones see below for single antibodies/MABTECH/3420-2APT/lots: 328, 340, 349, 354, 368, 449, 459
pre-coated plates with mAb 1-D1K
detection antibody/7-B6-1 (ALP)/ dilution 1:200
aCD3/clone CD3-2/1:1000

Antibodies for Multimer-Staining:
Reagent/ Clone/ Order No./ Manufacturer/ Dilution/ Lot(s)
CD3 APC-R700/ SK7/ 659119/ BD/ 1:100/ 9354980, 0351770, 1203086, 2270323, 3055422, 3265778
CD4 APC-Cy7/ OKT4/ 317418/ Biolegend/ 1:100/ B258921, B371717, B267977
CD16 APC-Cy7/ 3G8/ 561726/ BD/ 1:100/ 8333901, 2048998, 2237539
CD45RA BUV737/ HI100/ 612846/ BD/ 1:100/ 8173965, 9351398, 1105162, 2178611
CD8 BUV395/ RPA-T8/ 612914/ BD/ 1:50/ 8220831, 9259666, 2010508, 3044081
CD14 APC-Cy7/ MϕP9/ 561709/ BD/ 1:50/ 9009999, 2237607, 3212310, 2213975, 1060389, 2063515
CD19 APC-Cy7/ HIB19/ 302217/ Biolegend/ 1:50/ B279663, B361547
CD27 BV605/ L128/ 562656/ BD/ 1:50/ 9240362, 3184318, 1221678, 3018726, 3018727
CD57 BV510/ QA17A04/ 393314/ Biolegend/ 1:50/ B265765, B381252, B305922, B329650, B336705
CD197 PE-CF594/ 150503/ 562381/ BD/ 1:50/ 9270921, 8179961, 2048953, 2286100, 3068105
CD28 PerCP-Cy5.5/ CD28.2/ 560685/ BD/ 1:25/ 9319371, 3326026, 1293615, 3103933
CD279 BV650/ EH12/ 564104/ BD/ 0064420, 9336133, 8179961, 3180533, 2175198, 2312122
Viability eFluor780/ n.a./ 65-0865/ eBioscience/ 1:1667/ 2062571, 2469020, 2290916, 2469020, 1965980, 2851407
Streptavidin PE/ n.a./ 554061/ BD/ n.a./ 9123795
Streptavidin APC/ n.a./ 554067 BD/ n.a./ 3009576

Streptavidin BB515/ n.a./ 564453/ BD/ n.a./ 1340015
Streptavidin BV421/ n.a./ 563259/ BD/ n.a./ 3034768

Antibodies for TCF1 Multimer-Staining (only P01):
Reagent/ Clone/ Order No./ Manufacturer/ Dilution/ Lot
CD3 APC-R700/ SK7/ 659119/ BD/ 1:100/ 8234916, 9354980, 4221925, 1203086, 2270323, 3055422, 3265778
CD4 APC-Cy7/ OKT4/ 317418/ Biolegend/ 1:100/ B258921, B371717, B267977
CD16 APC-Cy7/ 3G8/ 561726/ BD/ 1:100/ 8333901, 3285439, 2048998, 2237539, 1102662
CD45RA BUV737/ HI100/ 612846/ BD/ 1:100/ 8173965, 9351398, 1105162, 2178611
CD8 BUV395/ RPA-T8/ 612914/ BD/ 1:50/ 8220831, 4046063, 2010508, 3044081, 0300778
CD14 APC-Cy7/ MϕP9/ 561709/ BD/ 1:50/ 8285873, 9009999, 4129471, 2213975, 1060389, 2063515
CD19 APC-Cy7/ HIB19/ 302217/ Biolegend/ 1:50/ B252246, B279663, B327112, B361547
CD27 BV605/ L128/ 562656/ BD/ 1:50/ 1221678, 3018726, 9309289, 7263551, 9240362, 4071606, 3018727
CD197 PE-CF594/ 150503/ 562381/ BD/ 1:50/ 8179961, 9270921, 3240340, 2048953, 2286100, 3068105, 1152617
CD28 PerCP-Cy5.5/ CD28.2/ 560685/ BD/ 1:25/ 7278835, 9319371, 3326026, 1293615, 129316, 3103933, 1011303
CD279 BV650/ EH12/ 564104/ BD/ 1:25/ 8316604, 9336133, 4030233, 3180533, 2175198, 2312122, 1060394
TCF1 Alexa488/ C63D9/ 6444S/ Cell Signaling/ 1:100/ 9, 10, 11, 13
CD127 BV711/ HIL-7R-M21/ 563165/ BD/ 1:50/ 8143567, 3201551, 9148848, 2325992

Antibodies for IC Staining
Reagent Clone Order No. Manufacturer
CD8 BB515/ RPA-T8/ 564527/ BD/ 1:33/ 8346690, 2070054, 0037189, 0342508
CD4 BV510/ OKT4/ 317444/ Biolegend/ 1:25/ B252920, B302135, B394263, B322925, B357641
CD16 APC-Cy7/ 3G8/ 561726/ BD/ 1:100/ 8333901, 2237539, 2048998, 2337539, 1102662
CD14 APC-Cy7/ MϕP9/ 561709/ BD/ 1:50/ 9009999, 2213975, 1060389, 2063515
CD19 APC-Cy7/ HIB19/ 302217/ Biolegend/ 1:50/ B252246, B327112, B361547, B279663
IFNγ PE-Cy7/ B27/ 557643/ BD/ 1:100/ 7202642, 9140862, 1229911, 2192264
TNF BV421/ Mab11/ 562783/ BD/ 1:167/ 8113890
Viability eFluor780 N/A 65-0865-14 eBioscience/ 1:1667/ 2290916, 2469020, 2062571, 1965980

Antibodies for IHC staining:
Reagent/ Clone/Lot/ Order No./ Manufacturer/ Dliution/ Lot
CD8/ SP16/ L535/ NBP2-26484/ Novus Biologicals/ 1:100/ G17662
CD3/ 2GV6/ G09184/ 05278422001/Roche Diagnostics/ ready-to-use/ G17662
MHC-I/ EPR1394Y/GR298302-7/ ab134189/ Abcam/ 1:500/ G17662
OmniMap anti-Rabbit HRP/ polyclonal/ G17662/ 0526967900/ Roche Diagnostics/ 1:500/ G17662

Validation

Commercial antibodies are validated by the manufacturer. BD biosciences and Novus bio performs quality control in line with ISO requirements including testing side by side with prior batches as a reference. Thermo Fischer, Biolegend and Cell signalling technologies test and certify antibodies using a flow cytometry assay. Commercial available antibodies were selected based on their antigen specificity and suggested application as described on the manufacturer`s website and data sheets. The antibody concentrations for staining were optimized by titrating down each reagent starting at the manufacturer`s recommendation. The optimal amounts of the reagents were defined by (i) minimal unspecific shift of the negative population and (ii) a maximal separation of the negative and positive population.

Eukaryotic cell lines

Policy information about cell lines and Sex and Gender in Research

Cell line source(s)

The Jurkat T cell line that expresses a luciferase reporter driven by an NFAT-response element is manufactured by Promega.

Authentication

Reauthentication of cell lines was performed by short tandem repeat (STR) profiling at ATCC and Eurofins.

Mycoplasma contamination

All used cell lines tested negative for mycoplasma contamination

Commonly misidentified lines
(See ICLAC register)

No commonly misidentified cell lines were used

Clinical data

Policy information about clinical studies
All manuscripts should comply with the ICMJE guidelines for publication of clinical research and a completed CONSORT checklist must be included with all submissions.

Clinical trial registration

NCT02316457

Study protocol

The study protocol for this clinical trial is not publically available, but the trial is described at ct.gov: https://clinicaltrials.gov/study/NCT02316457

Data collection

Clinical data was collected from three sites in Germany and one in Sweden. Study start was October 2016, with study completion May 2023, with patients followed up passively thereafter.

Outcomes

The primary objective was feasibility, safety and tolerability, with a secondary endpoint of vaccine-induced T cells responses

Plants

Seed stocks

Report on the source of all seed stocks or other plant material used. If applicable, state the seed stock centre and catalogue number. If plant specimens were collected from the field, describe the collection location, date and sampling procedures.

Novel plant genotypes

Describe the methods by which all novel plant genotypes were produced. This includes those generated by transgenic approaches, gene editing, chemical/radiation-based mutagenesis and hybridization. For transgenic lines, describe the transformation method, the number of independent lines analyzed and the generation upon which experiments were performed. For gene-edited lines, describe the editor used, the endogenous sequence targeted for editing, the targeting guide RNA sequence (if applicable) and how the editor was applied.

Authentication

Describe any authentication procedures for each seed stock used or novel genotype generated. Describe any experiments used to assess the effect of a mutation and, where applicable, how potential secondary effects (e.g. second site T-DNA insertions, mosiacism, off-target gene editing) were examined.

Flow Cytometry

Plots

Confirm that:

☒ The axis labels state the marker and fluorochrome used (e.g. CD4-FITC).

☒ The axis scales are clearly visible. Include numbers along axes only for bottom left plot of group (a 'group' is an analysis of identical markers).

☒ All plots are contour plots with outliers or pseudocolor plots.

☒ A numerical value for number of cells or percentage (with statistics) is provided.

Methodology

Sample preparation

For flow cytometry analysis PBMCs were used. PBMCs were isolated by ficoll density gradient from whole blood. For intracellular stains, cells were fixed and permeabilized using the eBioscience™ Foxp3/ Transcription Factor Staining Buffer Set.

Instrument

Flow Cytometry data was collected using the BD LSRFortessa™ SORP or BD FACSCanto™ II

Software

The BD FACSDiva™ software version 8.0.1 (FACSCanto II) or 8.0.2 (LSRFortessa) was used for data collection.
For data analysis, FlowJo software version 10 (BD).

Cell population abundance

N/A

Gating strategy

Respective gating strategies are detailed in the materials and methods section, in the supplementary information or in figure legends.

☒ Tick this box to confirm that a figure exemplifying the gating strategy is provided in the Supplementary Information.
